# An enantioselective artificial Suzukiase based on the biotin–streptavidin technology†
†Electronic supplementary information (ESI) available. See DOI: 10.1039/c5sc03116h


**DOI:** 10.1039/c5sc03116h

**Published:** 2015-10-19

**Authors:** Anamitra Chatterjee, Hendrik Mallin, Juliane Klehr, Jaicy Vallapurackal, Aaron D. Finke, Laura Vera, May Marsh, Thomas R. Ward

**Affiliations:** a Department of Chemistry , University of Basel , Spitalstrasse 51 , 4056 Basel , Switzerland . Email: thomas.ward@unibas.ch; b Swiss Light Source , Paul Scherrer Institute 5232 Villigen PSI , Switzerland

## Abstract

Introduction of a biotinylated monophosphine palladium complex within streptavidin affords an enantioselective artificial Suzukiase. Site-directed mutagenesis allowed the optimization of the activity and the enantioselectivity of this artificial metalloenzyme. A variety of atropisomeric biaryls were produced in good yields and up to 90% ee. The hybrid catalyst described herein shows comparable TOF to the previous aqueous-asymmetric Suzuki catalysts, and excellent stability under the reaction conditions to realize higher TON through longer reaction time.

## Abstract 2

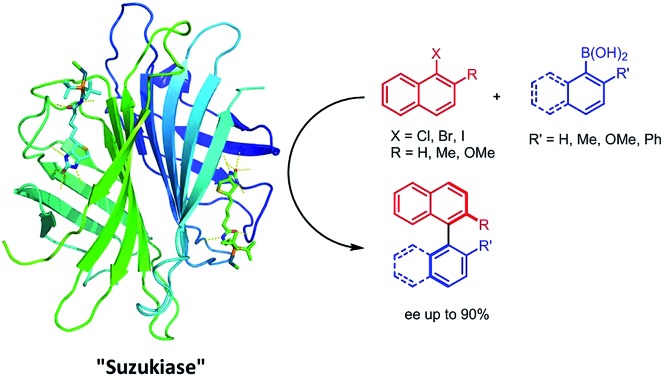
Introduction of a biotinylated monophosphine palladium complex within streptavidin affords an enantioselective artificial Suzukiase. Site-directed mutagenesis allowed the optimization of the activity and the enantioselectivity of this artificial metalloenzyme. A variety of atropisomeric biaryls were produced in good yields and up to 90% ee.

## Introduction

The palladium-catalyzed Suzuki–Miyaura cross-coupling reaction (SMC hereafter) of organic halides with boronic acids is one of the most versatile methods for the synthesis of biaryls.^1^–^3^ Such structural motifs are present in numerous agrochemicals, natural products, pharmaceuticals, and polymers.^4^,^5^ For this purpose, the SMC has been widely studied.^6^–^11^ More recently, the SMC has found applications in the context of chemical biology. Indeed, both reactants and products can be regarded as bio-orthogonal.^12^–^18^ In this promising context however, high catalyst loadings are routinely required with reactions performed in a biological environment.^12^,^13^,^15^,^17^,^19^–^27^

In stark contrast to the SMC, Nature relies on very different mechanisms to install (atropisomeric) C_aryl_–C_aryl_ bonds.^28^ Despite numerous reports on asymmetric SMC in organic media,^6^–^8^,^29^ only two reports describe enantioselective SMC in water.^30^,^31^ Uozumi reported on an heterogeneous SMC in water requiring a high catalyst loading (TON < 10, 94% ee at 80 °C).^30^ More recently, Kündig *et al.* reported an asymmetric SMC in an water–organic solvent mixture (TON = 17, up to 80% ee) at room temperature.^31^ To complement these efforts, we speculated that, thanks to their well-defined second coordination sphere, artificial metalloenzymes (ArMs hereafter) may offer a propitious environment to engineer an asymmetric “Suzukiase”-that is, an enzyme that catalyzes the SMC. ArMs result from the incorporation of an abiotic cofactor within a macromolecule (protein or oligonucleotide).^13^,^14^,^32^–^35^ In this context, Ueno and coworkers anchored a palladium moiety within a ferritin container to yield an artificial Suzukiase. The resulting artificial Suzukiase however did not outperform the free cofactor (no enantioselectivity, turnover frequency TOF: 3500 h^–1^, no TON mentioned).^13^ Inspired by a seminal contribution by Whitesides in 1978,^32^ we report herein our effort to engineer an asymmetric artificial Suzukiase based on the biotin–streptavidin technology.

## Results and discussion

**Scheme 1:**
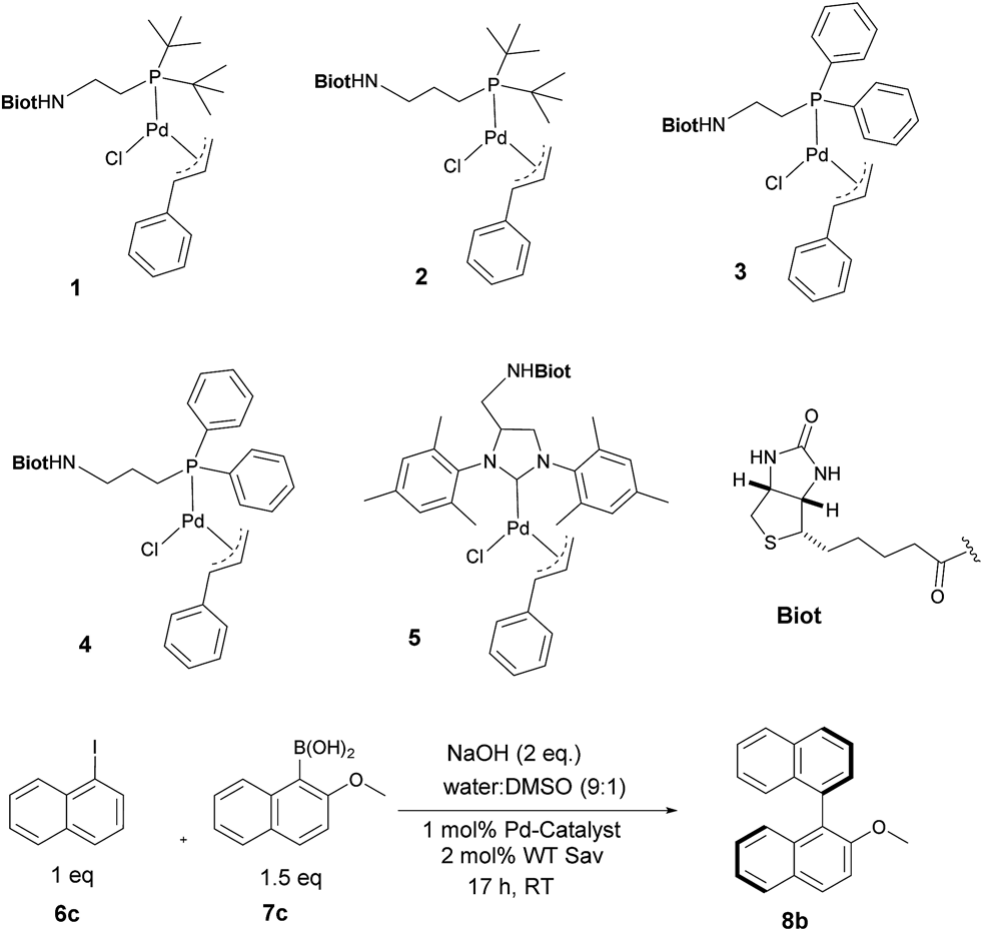
Biotinylated cofactors **1–5** tested in the presence of (strept)avidin as artificial Suzukiase for the synthesis of enantioenriched 2-methoxy-1,1′-binaphthyl **8b**.

With the aim of identifying suitable cross-coupling reaction conditions, we evaluated five different biotinylated catalyst precursors **1–5** in the presence of either avidin or streptavidin (Avi and Sav hereafter). For this purpose, the reaction of 1-iodonaphthalene **6c** with 2-methoxy-1-naphthaleneboronic acid **7c** was selected as a model system (Scheme 1). Initial experiments revealed that protonolysis of the boronic acid led to modest cross-coupling yields. Systematic variation of the base, pH, and organic co-solvent led to the identification of suitable reaction conditions: sodium hydroxide in DMSO : water (1 : 9) proved to be particularly effective in preventing protonolysis. However, competing deboronation required the use of excess boronic acid (1.5 equivalents *vs.* aryl halide) to achieve full conversion. Gel electrophoresis of the reaction mixture revealed that Sav remained largely tetrameric and active toward biotin binding even after an SMC performed at 50 °C (see ESI Fig. S3†).

**Fig. 1:**
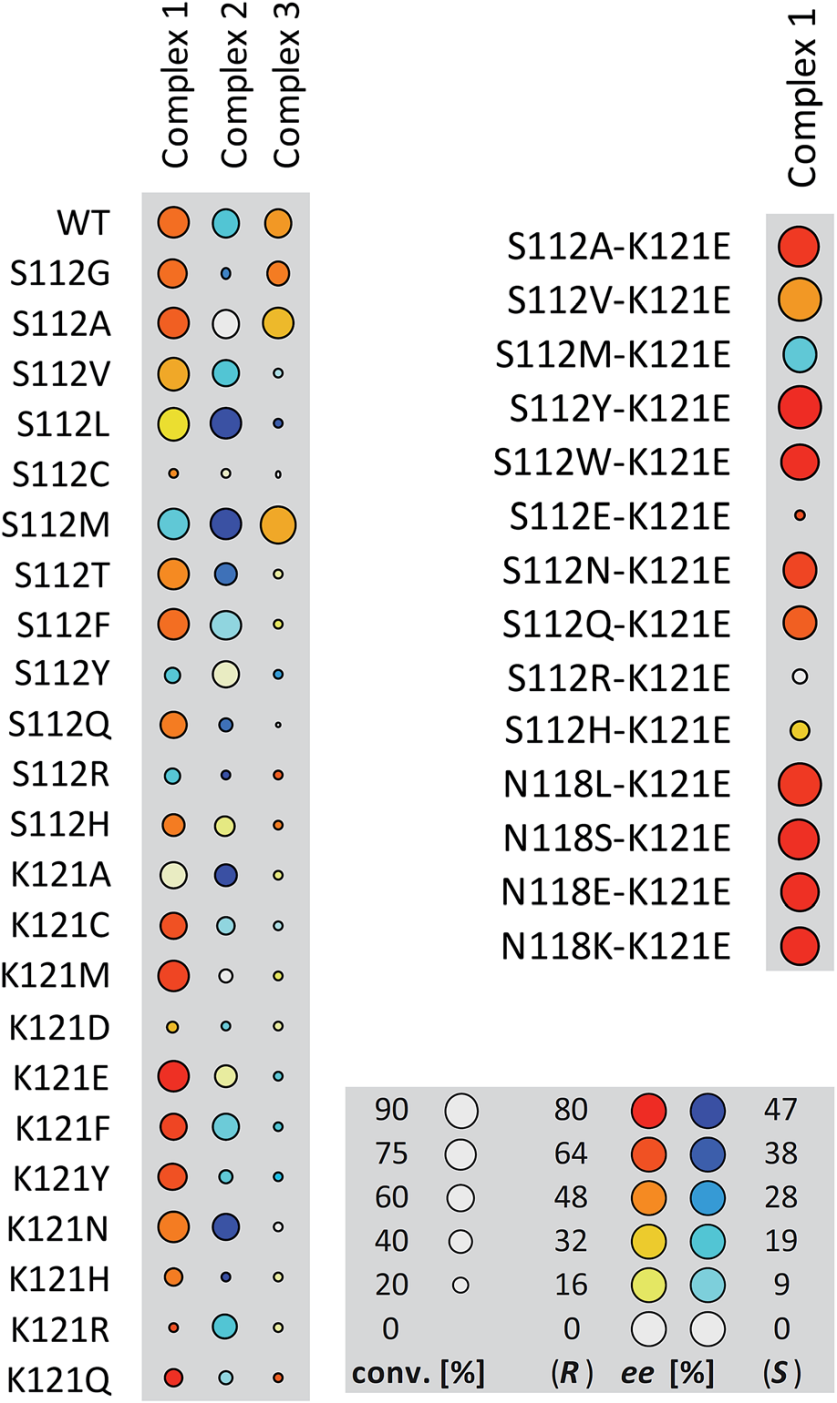
Fingerprint display of the results of the chemogenetic optimization for the synthesis of 2-methoxy-1,1′-binaphthyl **8b**. The size of the circles is proportional to the conversion, and the color codes the enantiomeric excess. Numerical results are collected Table 2 and in Table S1 (ESI†).

Having identified suitable reaction conditions, we screened biotinylated phosphine- and NHC ligands **1–5** combined with WT (strept)avidin (Table 1). This screening led to the identification of complexes **1**, **2**, and **3** as the most promising catalysts in combination with WT Sav. Despite the structural similarity between avidin and streptavidin, the result obtained with avidin was inferior to those obtained with Sav. We thus focused on Sav for further optimization studies. Past experience with other ArMs based on the biotin–streptavidin technology suggest that mutations at positions S112 and K121 (which lie closest to the biotinylated metal cofactor) offer a versatile means to rapidly optimize the ArM's performance. Complexes **1–3** were thus screened with a small library of Sav mutants. The results of the chemogenetic optimization for the synthesis of 2-methoxy-1,1′-binaphthyl **8b** are displayed as a fingerprint in Fig. 1, and selected results are collected in Table 2.

**Table 1:** Identification of the most suitable ligand for the synthesis of 2-methoxy-1,1′-binaphthyl **8b**^*a*^

Entry	Complex	Protein	ee^*b*^ [%]	TON
1	**1**	WT Sav	58 (*R*)	78
2	**2**	WT Sav	10 (*S*)	73
3	**3**	WT Sav	42 (*R*)	45
4	**4**	WT Sav	6 (*R*)	8
5	**5**	WT Sav	rac	<5
6	**1**	WT Avi	3 (*R*)	10

^*a*^Reactions were carried out with 50 mM substrate in a total reaction volume 0.2 mL using 1 mol% complex **1–5** (see ESI for experimental details).

^*b*^ee value determined by HPLC on a chiral stationary phase; absolute configuration assigned by comparison with literature data. WT = wild-type, Sav = streptavidin, Avi = avidin. All reactions were performed in duplicate: Δee = ±1%, Δconv. = ±5%.

**Table 2:** Selected results for genetic optimization of the artificial Suzukiase for the synthesis of 2-methoxy-1,1′-binaphthyl **8b**

Entry	Temp [°C]	Complex	Protein	ee [%]	TON
1	RT	**1**	—	rac	20
2	RT	**1**	WT	58 (*R*)	78
3	RT	**1**	K121E	76 (*R*)	50
4	RT	**1**	K121D	34 (*R*)	7
5	RT	**1**	K121Q	74 (*R*)	20
6	RT	**1**	K121M	67 (*R*)	59
7	RT	**1**	K121F	67 (*R*)	38
8	RT	**2**	K121A	47 (*S*)	32
9	RT	**2**	S112M	44 (*S*)	53
10	RT	**3**	S112M	38 (*R*)	78
11	RT	**3**	S112A	36 (*R*)	52
12	RT	**1**	S112M	14 (*S*)	58
13	RT	**1**	S112A	60 (*R*)	58
14	RT	**1**	N118K–K121E	74 (*R*)	73
15	RT	**1**	N118S–K121E	76 (*R*)	79
16	RT	**1**	N118E–K121E	76 (*R*)	75
17	RT	**1**	N118L–K121E	72 (*R*)	86
18	RT	**1**	S112W–K121E	76 (*R*)	64
19	RT	**1**	S112N–K121E	69 (*R*)	61
20	RT	**1**	S112A–K121E	70 (*R*)	80
21	RT	**1**	S112Y–K121E	80 (*R*)	90
22	RT	**1**	S112Y–K121E	80 (*R*)	160^*a*^
23	16	**1**	S112Y–K121E	84 (*R*)	50
24	4	**1**	S112Y–K121E	86 (*R*)	50^*b*^
25	4	**1**	S112Y–K121E	90 (*R*)	50^*c*^

^*a*^0.50 mol% catalyst loading, 0.25 mol% Sav (tetramer) loading.

^*b*^0.50 mol% catalyst loading, 0.25 mol% Sav (tetramer) loading, after 7 days.

^*c*^Preparative scale (100 μmol). All reactions were performed in duplicate: Δee = ±1%, Δconv. = ±5%.

From these data, the following features emerge:

(a) Compared to the free cofactor, higher conversions are observed with the artificial Suzukiase. This demonstrates that the Sav host protein exerts a beneficial influence on both the activity (*i.e.* protein accelerated catalysis)^36^ and the selectivity.

(b) The electron-donating properties and bulkiness of the phosphine play a key role in determining the activity of the corresponding artificial Suzukiase. Accordingly, the (*t*-Bu)_2_P-bearing catalysts outperform the (Ph)_2_P-systems. Strikingly, both ligands **1** and **3** (Table 2, entries 10–13) show similar activity in case of S112M and S112A mutants.

(c) The enantioenriched nature of the cross-coupled product **8b** strongly supports the hypothesis that the SMC is indeed catalysed by a homogeneous protein-embedded Pd-cofactor rather than Pd-nanoparticles.^12^,^25^,^26^,^37^

(d) Varying the spacer between the biotin anchor and the P(*t*-Bu)_2_ from ethyl to propyl affords opposite enantiomers of biaryl **8b** for a given Sav mutant. The best ees in favour of (*S*)-**8b** were obtained with **2**·Sav K121A and **2**·Sav S112M (Table 2, entries 8 and 9). These findings illustrate the versatility of chemogenetic optimization strategy: upon varying the length of the spacer the Pd-moiety experiences a very different second coordination sphere environment which is reflected in the enantioselectivity.

Having identified **1**·Sav K121E as a promising first generation Suzukiase (Table 2, entry 3), we screened double mutants bearing a glutamate at position 121 (Fig. 1 and Table 2, entries 14–21):

(a) The double mutant **1**·Sav S112A–K121E (Table 2, entry 20) gives better ee and TON than the single mutant Sav S112A (Table 2, entry 13). Likewise, **1**·Sav S112Y–K121E gave the highest turnover with good ee (Table 2, entry 22). This finding highlights the non-additive nature of multiple mutations.^38^,^39^ Decreasing the temperature to 4 °C leads to an improvement in enantioselectivity (86% ee, Table 2, entry 24) albeit at the cost of a slower rate.

(b) Increasing the ratio of complex **1***vs.* Sav tetramer (S112Y–K121E mutant) from one to four leads to a gradual erosion of enantioselectivity (from 80 to 69% ee, see Table S4, ESI†). This suggests that an empty biotin binding site adjacent to a complex **1** within Sav is favorable for selectivity.

(c) On a preparative scale, up to 90% ee (*R*)-**8b**, were obtained using 0.50 mol% catalyst loading and 0.25 mol% Sav (tetramer) loading at 4 °C (Table 2, entry 25).

(d) Upon decreasing the catalyst loading, a TON = 160 (TOF < 10 h^–1^) is obtained with a constant ee (Table 2 entries 21–22). This TON compares favourably with the other aqueous asymmetric homogeneous SMC catalysts (TON = 10,^30^ and 17 respectively^31^) compared to the achiral Suzukiase reported by Ueno^13^ however, the TOF is significantly lower (compare TOF 3500 h^–1^*vs.* <10 h^–1^).

**Fig. 2:**
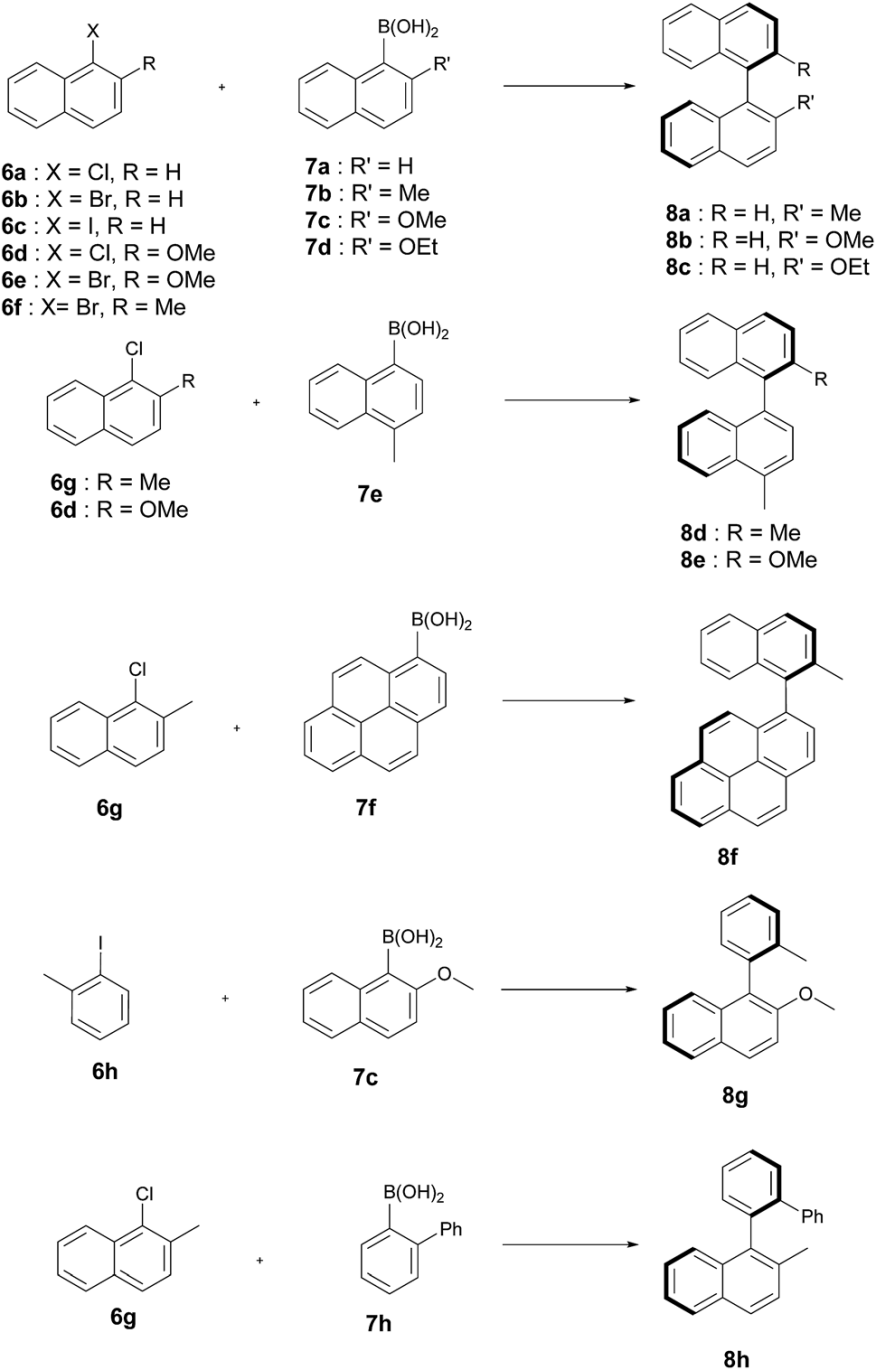
Substrates tested for the asymmetric Suzuki–Miyaura cross-coupling reaction catalysed by **1**·S112Y–K121E.

The artificial Suzukiase performed well on a range of hydrophobic substrates, leading to the following observations (Table 3, Fig. 2):

**Table 3:** Selected results for the SMC with **1**·S112Y–K121E Sav on a variety of substrates^*a*^

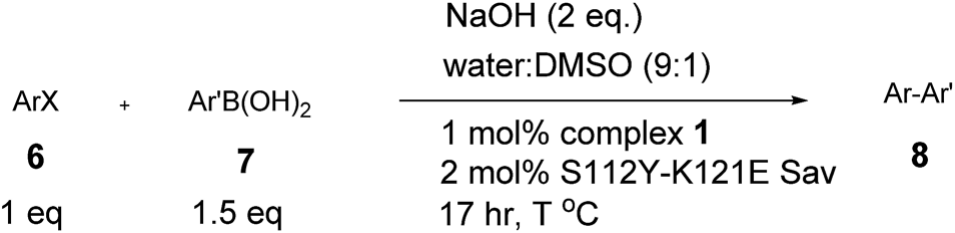						
Entry	ArX	Ar′B(OH)_2_	Product	Temp [°C]	ee [%]	TON
1	**6d**	**7a**	**8b**	RT	80 (*R*)	32
2	**6g**	**7e**	**8d**	50	80 (*S*)	20
3	**6d**	**7e**	**8e**	50	74 (*R*)	61
4	**6g**	**7f**	**8f**	50	64 (*S*)	50
5	**6g**	**7h**	**8h**	RT	65 (*R*)	29
6	**6a**	**7c**	**8b**	RT	80 (*R*)	7
7	**6b**	**7c**	**8b**	RT	80 (*R*)	63
8	**6c**	**7c**	**8b**	RT	80 (*R*)	80
9	**6f**	**7a**	**8a**	RT	69 (*S*)	64
10	**6b**	**7b**	**8a**	50	76 (*S*)	81
11	**6b**	**7b**	**8a**	4	87 (*S*)	8^*b*^
12	**6c**	**7d**	**8c**	RT	68 (*R*)	88
13	**6e**	**7a**	**8b**	RT	77 (*R*)	55
14	**6h**	**7c**	**8g**	RT	35 (*R*)	80

^*a*^All reactions were performed in duplicate: Δee = ±1%, Δconv. = ±5%. The absolute configuration of the product were assigned according to ref. 8. See ESI for experimental details.

^*b*^After 7 days.

(a) The nature of the aryl halide does not influence the enantioselectivity but to lower conversions on going from iodide to bromide to chloride **6a–c** (Table 3, entries 6–8).

(b) Substitution of the methoxy group by either a methyl and ethoxy group on the arylboronic acid (compare **7c** to **7b** and **7d**) leads lower enantioselectivities (Table 3, entries 7, 10 and 12). Depending on the nature of the *ortho*-substituent of the aryl moiety, the CIP priorities are inverted, yielding the opposite absolute configuration (*e.g.* compare **8b** and **8d**) for the same spacial arrangement. With the exception of the biphenyl-binaphthyl **8h**, all SMC products afford the same spacial arrangement using **1**·S112Y–K121E Sav. We speculate that upon binding to the palladium cofactor, the bulky biphenyl group **7h** forces the catalyst into a different second coordination sphere environment, leading to the opposite enantiomer (and spacial arrangement) of the coupled product.

(c) The system affords the highest enantioselectivity for binaphthyls or naphthyl-*o*-biphenyl products (albeit with the opposite chirality) in the presence of one smaller coupling partner, (*e.g.***6h**), the enantioselectivity is significantly lower.

## X-ray structure

**Fig. 3:**
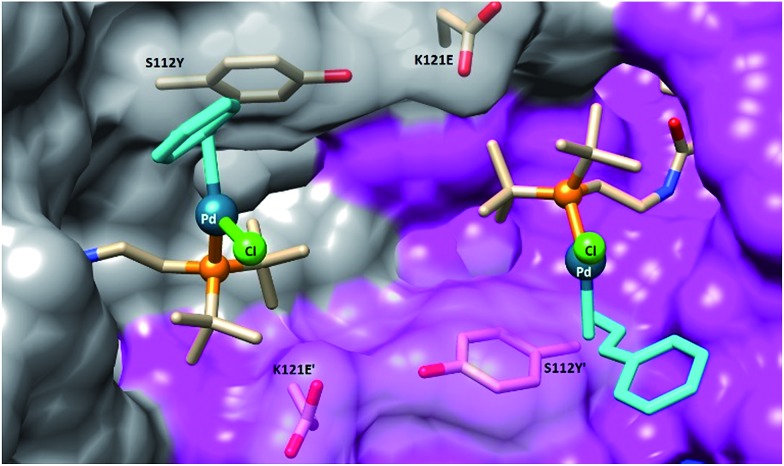
Close-up view of the structure of the artificial Suzukiase **1**·S112Y–K121E Sav (pdb code: 5CSE). The solvent-excluded surfaces of two symmetry-related Sav monomers are highlighted in grey and magenta respectively. The biotinylated Pd-cofactor **1** and the mutated residues S112Y and K121E are displayed as stick. No electron density for the cinnamyl-moiety (turquoise) could be detected and was thus modelled, minimizing steric-clashes.

To gain structural insight on the second coordination sphere around the Pd moiety, crystals of Sav S112Y–K121E were grown and soaked with a solution of **1** in DMSO (for details, see ESI†). The resulting crystals were subjected to X-ray diffraction at the beamline X06DA at the Swiss Light Source. In the resulting structure of complex **1**·S112Y–K121E Sav, there is strong residual electron density in the 2*F*_o_ – *F*_c_ difference map in the biotin-binding vestibule. The Pd atom was unambiguously assigned as a strong peak in the difference map (12*σ*) with a corresponding peak in the anomalous difference map (8*σ*). The density map clearly highlights the presence of the phosphine ligand, the palladium, and the chloride (see ESI Fig. S5†). However, the cinnamyl ligand could not be resolved, likely due to disorder caused by ligand fluxionality. The amide linker exhibits two H-bonding interactions with the protein backbone: the amide nitrogen exhibits an H-bonding interaction with the side-chain of S88, and the carbonyl oxygen exhibits an H-bonding interaction with the backbone nitrogen of N49. The palladium atoms of two symmetry related-cofactors (face-to-face) are separated by 11.7 Å. The palladium was found in the vicinity of the O atoms of S112Y and K121E (Pd···O distance 6.4 Å and 7.1 Å respectively). The average B-factor of Pd is 36.10, suggesting that the complex may be fluxional or located in a shallow energy minimum (Fig. 3).

In the synthesis of chiral biaryls by the SMC, enantioselectivity is dictated by the conformation of the Pd intermediate just prior to reductive elimination.^40^,^43^ In previous DFT studies of enantioselective reductive eliminations in the SMC, it was shown that weak interactions between substrates and substrate–ligand interactions were largely responsible for biasing certain conformations and thus leading to enantioinduction.^6^,^40^,^44^ By contrast, in the **1**·Sav system, it is more likely that the origin of the enantioselectivity observed is from the outer-sphere ligand effect of the protein. Inspection of the X-ray structure of the active site of **1**·S112Y–K121E shows that the Pd-phosphine ligand is effectively “locked” into place by the steric environment of the Sav protein, which is evidenced by the discrete atomic position of the Pd and P and their relatively low B-factors despite proximity to the solvent pocket. The restricted conformational space around the Pd atom limits certain conformations. The nearby mutated residues play an additional role. In particular, mutation of S112 to the larger tyrosine provides a significant steric barrier on the lower face of the Pd atom and is most likely responsible for the modest improvement in enantioselectivity upon mutation. Furthermore, the conformation of Y112 is effectively locked in place by an H-bonding interaction of the sidechain oxygen with the backbone carbonyl of E121 (distance 2.88 Å), which would not be the case with the similarly large S112W mutation (Table 2, entry 18). Further X-ray studies of **1**·Sav with various mutations are currently underway.

It should be emphasized however that, while the X-ray structure sheds light on the position of the biotinylated catalyst precursor, we cannot exclude that the loaded catalyst, bearing the two bulky aromatic coupling partners, adopts a different position within the biotin-binding vestibule. As demonstrated recently for an artificial imine reductase, QM-MM studies may shed light on the enantioselection mechanism and the precise geometry of the transition state.^41^ In this context, reducing the cofactor flexibility by introducing stabilizing interactions with the protein has proven useful.^42^

## Conclusions

In summary, incorporation of an electron-rich phosphino–palladium moiety within Sav affords an artificial Suzukiase for the synthesis of enantioenriched binaphthyls (up to 90% ee and 50 TONs for 2-methoxy-binaphthyl **8b**). While it is straightforward to identify aminoacids close to the cofactor, it is remains extremely challenging to predict or rationalize why a given mutation leads to an improvement in catalytic performance. The chemogenetic optimization strategy allows to address this challenge with a limited effort: combining a small library of *m* mutants with a small library of *n* biotinylated ligands affords an *m*·*n* diversity matrix. Current efforts are aimed at performing catalytic asymmetric SMC with artificial Suzukiases *in vivo*.

## Supplementary Material

Supplementary information
