# Supplementary material for: An enantioselective artificial Suzukiase based on the biotin–streptavidin technology
Source: Chem Sci. 2015 Oct 19;7(1):673–7. doi: 10.1039/c5sc03116h (PMC5953008; doi:10.1039/c5sc03116h)
Supplement: Supplementary file 1 [file SC-007-C5SC03116H-s001.pdf]

Electronic Supplementary Material (ESI) for Chemical Science.
This journal is © The Royal Society of Chemistry 2015

Supporting Information for

Enantioselective Artificial Suzukiase for the Synthesis of Axially Chiral Biaryl Compounds

Anamitra Chatterjee, Hendrik Mallin, Juliane Klehr, Jaicy Vallapurackal, Aaron D. Finke, Laura Vera, May Marsh and Thomas R. Ward*

General: All Chemicals were of reagent grade and used as commercially purchased without further purification. ^{1}H and ^{13}C spectra were recorded on a Bruker 400 MHz. Chemical shifts are reported in ppm (parts per million). Signals are quoted as s (singlet), d (doublet), t (triplet), brs (broad) and m (multiplet). Analysis of the catalytic runs were performed on an Agilent 1100 normal phase HPLC with an analytical Chiracel OJ-H column (5μm, 250 x 4.6 mm) of Daicel Chemical Ind.

Protein expression and purification:

Native, egg avidin was obtained from Belovo and used without further purification. Recombinant streptavidin isoforms were engineered, expressed, purified and quantified as previously described. [1]

Synthesis of biotinylated catalysts:

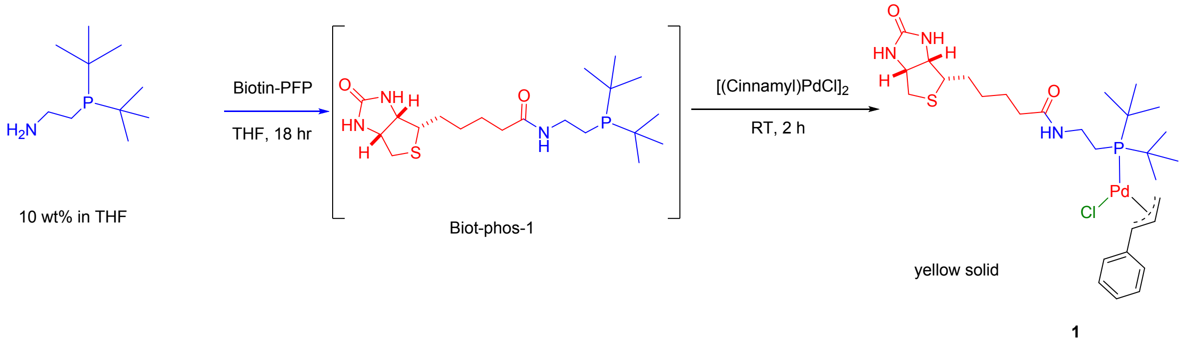

Scheme 1. Synthesis of complex **1**

Complex 1: The Biotin-PFP ester [2] (200 mg, 0.49 mmol) was set in a schlenk flask. The flask was evacuated and backfilled with nitrogen three times and dry THF was added. The 2-(Di-*t*-butylphosphino)ethylamine (10 wt. % in THF, 1 ml, 0.49 mmol) was added slowly to the suspension. The reaction mixture was stirred at room temperature overnight (18-20 h). Then [Pd(cinnamyl)(μ-Cl)]$_2$ (130 mg, 0.25 mmol) was added to the clear solution and stirred for 2 h at

room temperature. After this time, THF was removed under reduced pressure and the crude product was purified by flash chromatography on silica gel (MeOH : CH_2Cl_2 5:95) to afford the complex **1** as yellow solid (240 mg, 74%).

^{1}H NMR (400 MHz, $CDCl_3$): δ = 7.49 (d, J = 7.7 Hz, 2H), 7.42 – 7.29 (m, 3H), 5.88 – 5.72 (m, 1H), 5.23 (dd, J = 13.2, 9.2 Hz, 1H), 5.07 (s, 1H), 4.44 – 4.32 (m, 1H), 4.23 (dd, J = 14.6, 7.5 Hz, 1H), 3.97 (s, 1H), 3.59 (s, 2H), 3.13 (s, 1H), 2.81 – 2.62 (m, 1H), 2.22 – 2.05 (m, 2H), 1.76 – 1.53 (m, 6H), 1.54 – 1.15 (m, 24H).

^{13}C NMR (100 MHz, $CDCl_3$): δ = 173.5, 164.1, 136.5, 128.7, 128.2, 127.9, 108.7, 101.8, 61.7, 60.0, 56.0, 48.1, 40.8, 37.1, 35.8, 30.0, 28.1, 27.8, 25.6, 21.5, 21.4.

^{31}P NMR (162 MHz, $CDCl_3$): δ = 62.3.

HRMS [ESI(+)TOF]: calculated for $C_{29}H_{47}N_3O_2PPdS$ [M-Cl]+ 638.2156; found 638.2172.

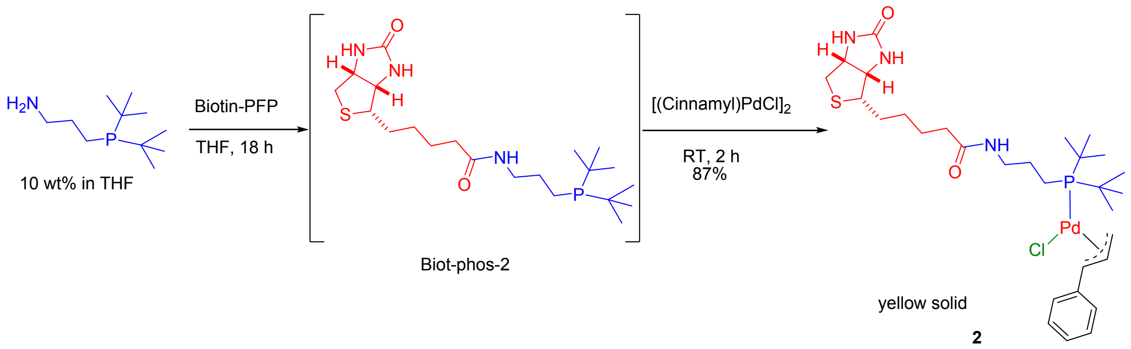

Scheme 2. Synthesis of complex **2**

Complex 2: Complex **2** was synthesized following the same procedure used for the synthesis of Complex **1**, using Biotin-PFP ester (90 mg, 0.49 mmol), 3-(Di-*t*-butylphosphino)propylamine (10 wt. % in THF, 0.5 ml, 0.22 mmol) and [Pd(cinnamyl)(μ-Cl)]$_2$ (67 mg, 0.12 mmol). The crude product was purified on silica gel by 5% MeOH/CH_2Cl_2 to yield the title product as yellow solid (140 mg, 87%).

^{1}H NMR (400 MHz, $CDCl_3$): δ = 7.58 – 7.30 (m, 5H), 5.89 – 5.67 (m, 1H), 5.38 – 5.24 (m, 1H), 4.88 (d, J = 20.4 Hz, 1H), 4.40 – 4.31 (m, 1H), 4.25 – 4.05 (m, 1H), 3.93 (dd, J = 6.9, 5.4 Hz, 1H), 3.28 (s, 2H), 3.15 (s, 1H), 2.92 (s, 1H), 2.77 – 2.57 (m, 2H), 2.17 (t, J = 7.3 Hz, 2H), 2.12 – 1.82 (m, 4H), 1.79 – 1.46 (m, 6H), 1.44 – 1.11 (m, 20H).

^{13}C NMR (100 MHz, $CDCl_3$): δ = 174.3, 163.6, 136.5, 129.3, 128.8, 128.2, 127.9, 108.9, 103.2, 61.6, 59.9, 55.8, 40.6, 40.5, 35.8, 34.9, 34.8, 30.1, 30.0, 27.8, 25.7, 18.5.

^{31}P NMR (162 MHz, $CDCl_3$): δ = 66.7.

HRMS [ESI(+)TOF]: calculated for $C_{30}H_{49}N_3O_2PPdS$ [M-Cl]+ 652.2312; found 652.2325.

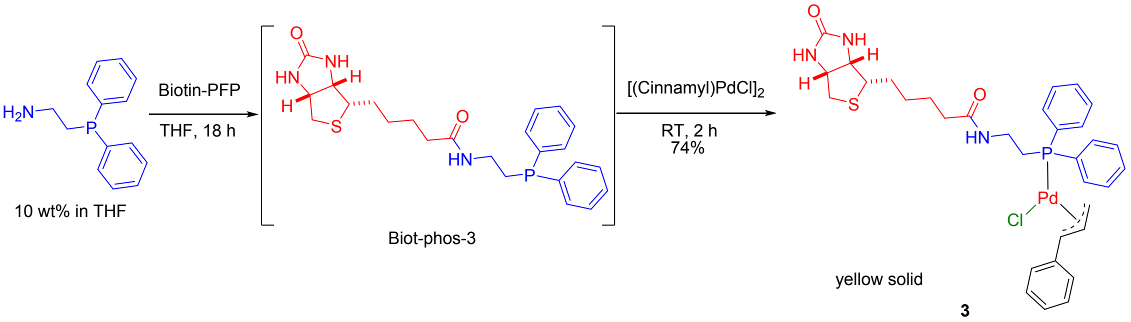

Scheme 3. Synthesis of complex **3**

Complex 3: Complex **3** was synthesized following the same procedure used for the synthesis of Complex **1**, using Biotin-PFP ester (181 mg, 0.44 mmol), 3-(Di-t butylphosphino)propylamine (10 wt. % in THF, 85 µl, 0.22 mmol) and $[Pd(cinnamyl)(\mu\text{-}Cl)]_2$ (67 mg, 0.12 mmol). The crude product was purified on silica gel by 5% MeOH/CH_2Cl_2 to yield the title product as yellow solid (140 mg, 87%).

^{1}H NMR (400 MHz, $CDCl_3$): δ = 7.63 – 7.28 (m, 18H), 6.18 (s, 1H), 5.99 (dt, J = 13.2, 9.4 Hz, 2H), 5.40 – 5.19 (m, 2H), 4.44 – 4.31 (m, 1H), 4.29 – 4.17 (m, 1H), 3.49 (dd, J = 16.3, 6.0 Hz, 2H), 3.31 – 2.94 (m, 1H), 2.76 (s, 2H), 1.93 (t, J = 7.3 Hz, 2H), 1.75 (s, 2H), 1.63 – 1.36 (m, 3H), 1.30 (d, J = 6.5 Hz, 2H).

^{13}C NMR (100 MHz, $CDCl_3$): δ = 173.6, 163.7, 136.2, 132.8, 132.7, 132.6, 130.6, 129.0, 128.9, 128.5, 127.9, 111.5, 100.7, 100.4, 61.4, 59.8, 56.5, 35.6, 28.1, 27.9, 27.8, 27.6, 25.4.

^{31}P NMR (162 MHz, $CDCl_3$): δ = 18.4.

HRMS [ESI(+)TOF]: calculated for $C_{33}H_{39}N_3O_2PPdS$ [M-Cl]+ 678.1530; found 678.1551.

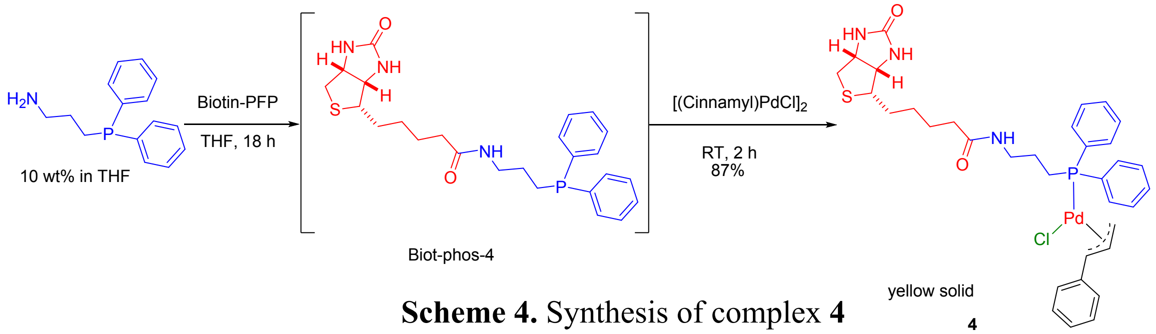

Scheme 4. Synthesis of complex **4**

Complex 4: Complex **4** was synthesized following the same procedure used for the synthesis of Complex **1**, using Biotin-PFP ester (123 mg, 0.3 mmol), 3-(Di-*t*-butylphosphino)propylamine (10 wt. % in THF, 97 mg, 0.4 mmol) and $[Pd(cinnamyl)(\mu\text{-}Cl)]_2$ (67 mg, 0.12 mmol). The crude

product was purified on silica gel by 5% MeOH/CH_2Cl_2 to yield the title product as yellow solid (140 mg, 87%).

^{1}H NMR (400 MHz, $CDCl_3$): δ = 7.63 – 7.29 (m, 15H), 6.19 – 5.83 (m, 3H), 5.43 – 5.26 (m, 1H), 5.16 (s, 1H), 4.34 – 4.19 (m, 1H), 4.20 – 4.06 (m, 1H), 3.26 (s, 4H), 2.76 – 2.36 (m, 3H), 2.18 (t, J = 7.0 Hz, 2H), 1.87 – 1.51 (m, 8H), 1.35 (br, 2H).

^{13}C NMR (100 MHz, $CDCl_3$): δ = 173.9, 163.5, 137.0, 136.4, 136.3, 132.7, 130.6, 129.2, 128.9, 128.8, 128.5, 128.3, 127.8, 127.7, 111.1, 111.0, 101.0, 100.0, 61.1, 59.6, 54.5, 39.7, 35.8, 27.7, 27.4, 25.5, 25.0, 24.8, 24.1.

^{31}P NMR (162 MHz, $CDCl_3$): δ = 24.6.

HRMS [ESI(+)TOF]: calculated for $C_{34}H_{41}N_3O_2PPdS$ [M-Cl]+ 692.1686; found 692.1703.

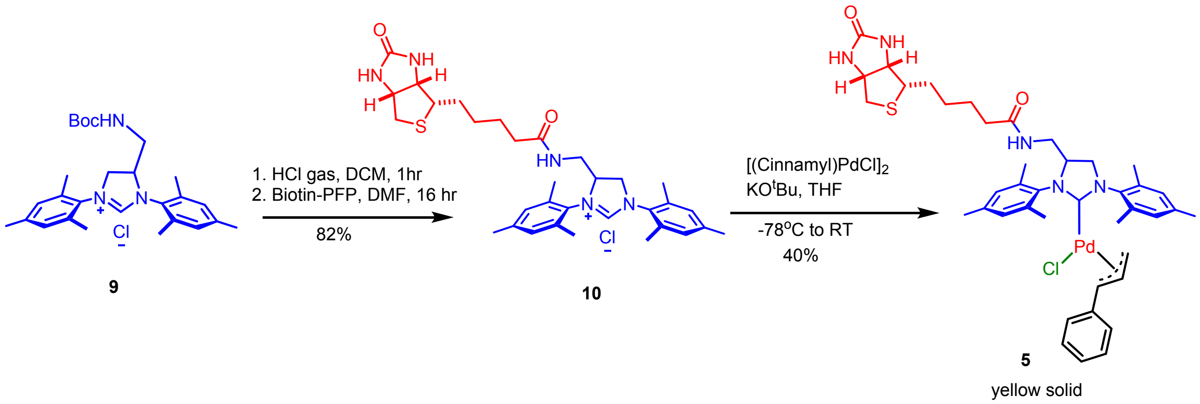

Scheme 5. Synthesis of complex **5**

Biot-NHC (10): Complex **9** was synthesized according to literature procedure.[3] Complex **9** (236 mg, 0.5 mmol) was dissolved in CH_2Cl_2 (2 ml) and HCl gas was bubbled through the solution for 1 hour at room temperature. The gaseous HCl was generated by the dropwise addition of concentrated H_2SO_4 to NH_4Cl. The solution was stirred for 2 hours at room temperature. The deprotection step was monitored by thin-layer chromatography (MeOH/CH_2Cl_2 1:9). The solvent was evaporated and the resulting solid was dissolved in DMF (2 ml). Biotin pentafluorophenol (164 mg, 0.4 mmol) and Et_3N (1.4 ml, 10 mmol) was added to the solution and stirred for 16 h at room temperature. The solvent was removed at reduced pressure. The crude product was purified on silica gel by 10% MeOH/CH_2Cl_2 to yield compound **10** as white solid (197 mg, 82%).

^{1}H NMR (400 MHz, CD_2Cl_2): δ = 9.38 (d, J = 5.3 Hz, 1H), 8.86 (br, 1H), 7.05 – 6.90 (m, 4H), 6.72 (s, 1H), 6.32 (s, 1H), 5.16 (br, 1H), 4.52 (s, 2H), 4.31 (s, 1H), 4.11 (s, 1H), 3.67 (d, J = 42.9 Hz, 1H), 3.32 (d, J = 9.9 Hz, 1H), 3.27 – 3.13 (m, 1H), 2.79 – 2.69 (m, 1H), 2.62 (dd, J = 19.7, 12.8 Hz, 1H), 2.50 – 2.21 (m, 20H), 2.12 (s, 2H), 1.71 – 1.38 (m, 4H).

^{13}C NMR (100 MHz, CD_2Cl_2): δ = 174.7, 160.2, 140.9, 140.7, 135.9, 135.8, 135.7, 130.8, 130.7, 130.4, 130.2, 129.7, 129.6, 62.8, 62.2, 62.0, 60.5, 56.3, 56.2, 56.1, 46.2, 40.9, 35.8, 28.5, 28.4, 25.9, 25.6, 21.3, 21.2, 19.2, 18.7, 8.8.

HRMS [ESI(+)TOF]: calculated for $C_{32}H_{44}N_5O_2S$ [M-Cl]+ 562.3215; found 562.3212.

Complex 5: Compound **10** (201 mg, 0.34 mmol), [Pd(cinnamyl)(μ-Cl)]$_2$ (82 mg, 0.16 mmol) and KO^{t}Bu (38 mg, 0.34 mmol) were set in a schlenk flask. The flask was evacuated and backfilled with nitrogen three times, then 9 ml dry THF was added. The flask was cooled at -78 ^oC for 1h and then warmed to RT and stirred for another 1h. The solvent was removed under reduced pressure. The crude product was purified on silica gel using 10% MeOH/CH_2Cl_2 to yield compound **5** as yellow solid (52 mg, 40%).

^1H NMR (400 MHz, CD_2Cl_2): δ = 7.36 – 6.90 (m, 9H), 6.49 (s, 1H), 5.83 (s, 1H), 4.94 – 4.68 (m, 1H), 4.54 – 4.31 (m, 2H), 4.17 (d, J = 11.8 Hz, 2H), 4.01 (t, J = 10.7 Hz, 1H), 3.91 – 3.76 (m, 1H), 3.50 (br, 1H), 3.36 – 3.07 (m, 3H), 2.88 (m, 2H), 2.65 (d, J = 8.4 Hz, 1H), 2.55 – 2.20 (m, 18H), 2.15 – 1.98 (m, 3H), 1.75 – 1.42 (m, 6H).

^{13}C NMR (100 MHz, CD_2Cl_2): δ = 209.8, 168.0, 162.1, 145.7, 136.4, 135.1, 127.7, 126.9, 125.9, 125.3, 122.9, 107.6, 89.8, 61.6, 60.3, 58.6, 54.3, 52.8, 45.0, 31.6, 27.1, 27.0, 26.8, 25.3, 24.2, 23.3, 22.5.

HRMS [ESI(+)TOF]: calculated for $C_{41}H_{52}N_5O_2PdS$ [M-Cl]+ 784.2876; found 784.2890.

General procedure for Catalysis on an analytical scale:

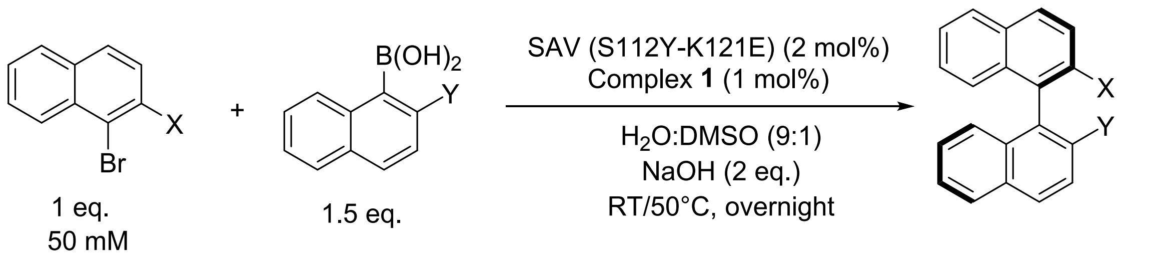

Apart from the extraction and the purification of the products everything was carried out in the glove-box using nitrogen-flushed solvents.

A stock solution of Sav (0.20 μmol, 0.02 eq.) in mQ water and the catalyst (0.10 μmol, 0.01 eq.) in DMSO were prepared, added together and stirred for ten minutes at room temperature (25 ^oC). Stock solutions of NaOH (20 μmol, 2.00 eq.) in mQ water, aryl-halide (10 μmol, 1.00 eq.) and boronic acid (15 μmol, 1.50 eq.) in DMSO were prepared, added to the mixture and stirred

overnight (17 h). The reactions were performed with iodonaphthalene, bromonaphthalene and chloronaphthalene derivatives at two different temperatures (25 °C and 50 °C) in the scale of 50 mM in 200 µL total volume.

Upon completion of the reaction, the reaction vessel was removed from the glove box and 1,2,4-trimethylbenzene (100 µL, 10.0 mM in TBME, as internal standard) was added. It was then extracted with TBME (700 µL), dried over Na_2SO_4. The conversion and enantiomeric excess were determined by chiral HPLC using an Analytical Chiracel OJ-H column (4.6 mm x 250 mmL, particle size 5µm) and a hexane / isopropanol mixture as eluent.

Reactions on 100 µmol scale (preparative scale), general procedure:

A stock solution of Sav (1.00 µmol, 0.01 eq.) in mQ water and the catalyst (0.50 µmol, 0.005 eq.) in DMSO were prepared, added together and stirred for ten minutes at 4 ^{o}C. Stock solutions of NaOH (200 µmol, 2.00 eq.) in mQ water, 1-Iodonaphthalene (15 µl, 100 µmol) and 2-methoxy-1-naphthaleneboronic acid (30 mg, 150 µmol) in DMSO were prepared, added to the mixture and stirred for 7 days at 4 ^{o}C in a sealed vial under nitrogen (2 mL total reaction volume). The reaction mixture was extracted with EtOAc (3 x 10 ml). After drying of the organic phases and removal of volatiles, the crude product was purified by column chromatography over silica gel with EtOAc/cyclohexane (1 : 10). The product was obtained as colourless oil (7 mg, 25 %, 90 % ee). See SI Figure 1 and 2 for ^{1}H NMR and chiral phase HPLC-analysis.

^{1}H NMR (400 MHz, $CDCl_3$) δ 7.99 (d, J = 9.0 Hz, 1H), 7.96 (dd, J = 8.2, 4.1 Hz, 2H), 7.88 (d, J = 8.2 Hz, 1H), 7.63 (dd, J = 8.3, 7.0 Hz, 1H), 7.50 – 7.42 (m, 3H), 7.37 – 7.27 (m, 3H), 7.25 – 7.21 (m, 1H), 7.17 (dd, J = 9.1, 0.6 Hz, 1H), 3.77 (s, 3H).

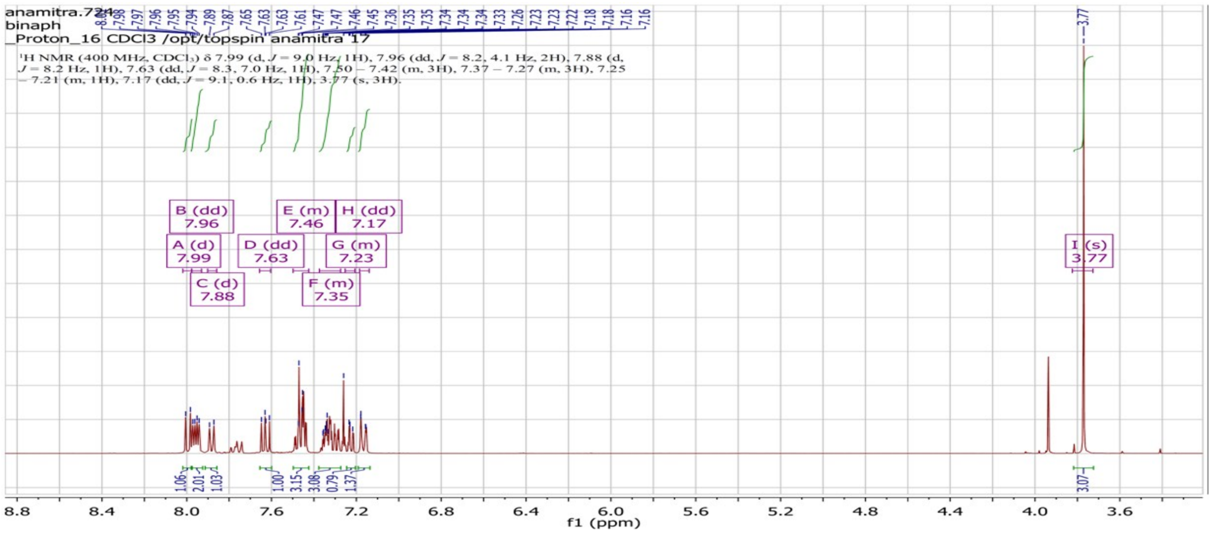

Supporting Figure 1. ^{1}H NMR of isolated product **8b**.

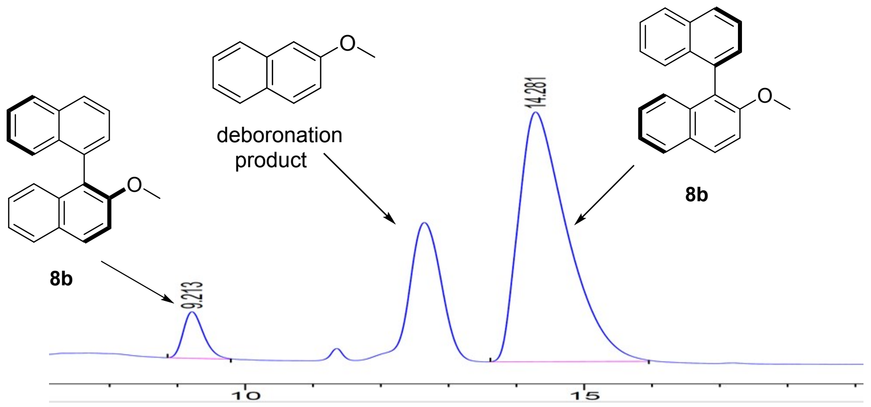

Peak #	RetTime [min]	Type	Width [min]	Area [mAU*s]	Height [mAU]	Area %
1	9.213	PM	0.2903	300.23758	17.23774	5.2641
2	14.281	BB	0.7412	5403.24658	107.72026	94.7359

Supporting Figure 2. Chiral phase HPLC data of isolated product.

SDS-Page Analysis of reaction mixtures:

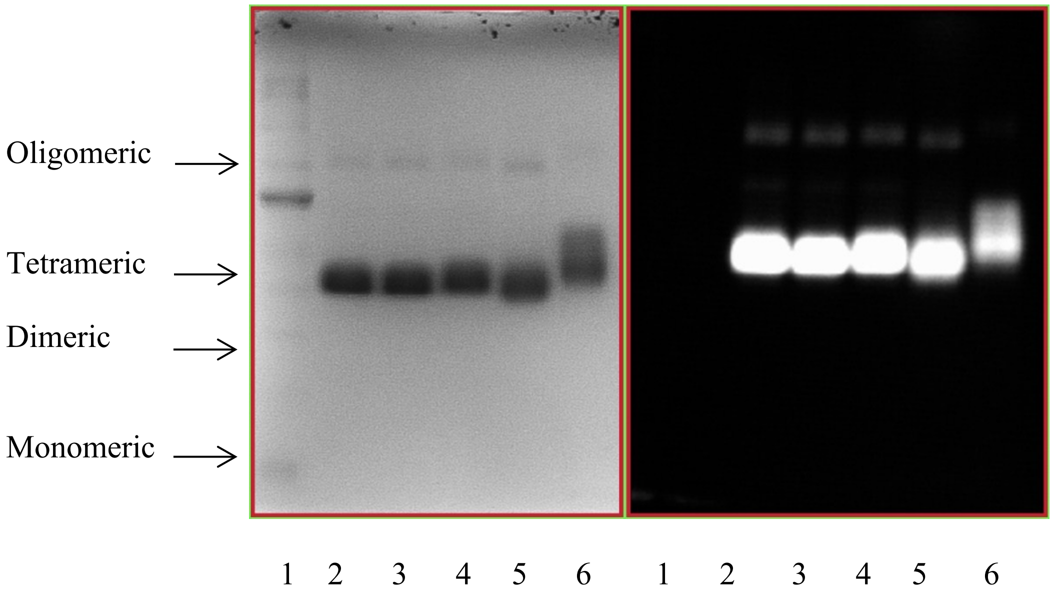

1 = protein ladder
2 = S112Y-K121E
3 = S112Y-K121E + Catalyst +DMSO
4 = S112Y-K121E + Catalyst +DMSO + NaOH
5 = S112Y-K121E + Catalyst +DMSO + NaOH + substrate
6 = S112Y-K121E + Catalyst +DMSO + NaOH + substrate + 24 hr

Supporting Figure 3. SDS-Page Gels. Right - B_4F Stain, Left - Commassie Stain

It is well established that non-denatured, active streptavidin can be detected by SDS-PAGE using biotinylated fluorescein as a specific marker (right side of SI Fig. 3).[4,5] The same SDS-PAGE gels can also be stained with comassie-blue to visualize all proteins (left side of Fig. 4). Under non-denaturing conditions, streptavidin migrates primarily as an active tetramer, although the protein also has a tendency to aggregate to form oligomers (in some cases, dimers and monomers of streptavidin are also commonly detected as minor, active species). In the present study, under catalytic conditions (including 75 equivalents of boronic acid and 100 equivalents of NaOH), the reaction mixture formed a suspension; upon microcentrifugation at 14’000 rpm, this suspension could be separated into a pellet and a clear supernatant. The supernatant contained active streptavidin (Non-denatured), which was binds biotinylated fluorescein. The migrating properties of the protein in this supernatant were similar to a Sav control (Non-denatured S112Y-K121E), with the corresponding expected molecular weight (Protein Ladder).

These figures show that as each solvent and compound is added to the reaction, the Sav isoform remains largely as the active tetramer or oligomer. The isolated precipitate reactions is largely starting material and product.

Ee and conversion over the time course of the reaction:

Following the general protocol for catalysis, 13 individual vials were prepared. The reactions were extracted at the indicated time points as described for the reactions on an analytical scale and analysed by HPLC.

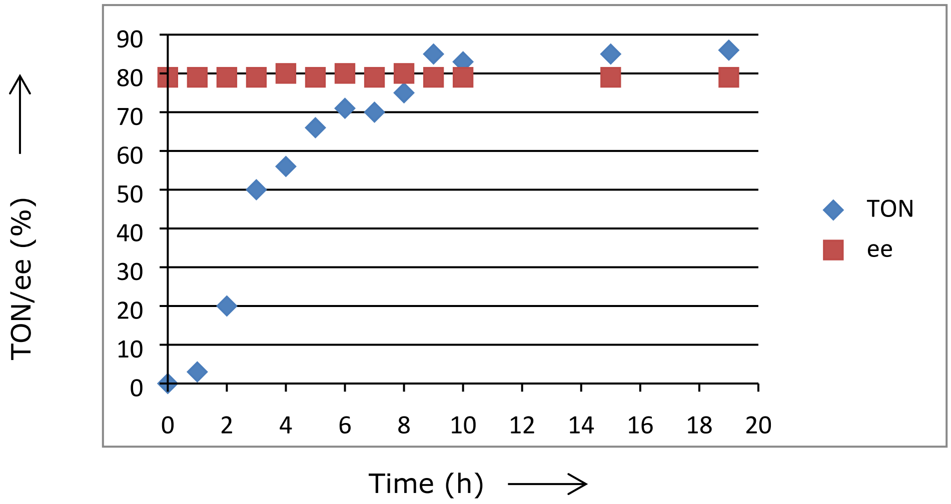

Supporting Figure 4. Conversion over the time course of the reaction

Supporting Table 1. Complete list of catalytic experiments carried out for the chemogenetic optimization (see fingerprint display, Figure 1) with complex **1**, **2** and **3**. Enantiomeric excess > 0 corresponds to [*S*] > [*R*]; enantiomeric excess < 0 [*R*] > [*S*]

Complex	Mutant	TON	ee [%]
**1**	WT	55, 57, 53	-57, -57, -56
**1**	S112Y	13,16	+16,+15
**1**	S112L	57, 60	-26, -26
**1**	S112V	59, 60	-38,-37
**1**	S112F	54, 56	-54, -54
**1**	S112M	57, 59	+14, +14
**1**	S112T	55, 60	-49, -49
**1**	S112Q	43, 42	-50, -50
**1**	S112C	<5, <5	-48, -47
**1**	S112R	13, 17	+16, +15
**1**	S112G	46, 50	-56, -56
**1**	S112H	24, 28	-50, -51
**1**	S112A	57, 59	-60, -60
**1**	K121R	6, 8	-62, -63
**1**	K121E	52, 48	-76,-75
**1**	K121D	7, 10	-34,-33
**1**	K121A	37, 41	-4, -5
**1**	K121Y	44, 49	-64, -65

**1**	K121F	36, 40	-67, -67
**1**	K121M	59, 58	-67, -67
**1**	K121N	52, 50	-50, -50
**1**	K121H	17, 19	-53, -54
**1**	K121C	35, 39	-62, -61
**1**	-	22, 19	rac, rac
**2**	WT	44, 45, 42	+19, +18, +19
**2**	S112Y	37, 38	-3, -3
**2**	S112L	53, 50	+45, +45
**2**	S112V	41, 37	+18, +18
**2**	S112F	48, 50	+7, +7
**2**	S112M	50, 55	+44, +43
**2**	S112T	23, 29	+33, +34
**2**	S112Q	7, 10	+33, +32
**2**	S112C	<5, <5	-3, -4
**2**	S112R	<5, <5	+44, +45
**2**	S112G	6, 7	+31, +32
**2**	S112H	21, 26	-11, -12
**2**	S112A	44, 48	rac, rac
**2**	K121R	35, 31	+19, +19
**2**	K121E	33, 28	-9, -9
**2**	K121D	<5, <5	+12, +11

**2**	K121A	34, 30	+47, +46
**2**	K121Y	8, 10	+14, +15
**2**	K121F	37, 33	+11, +12
**2**	K121M	14, 10	rac, rac
**2**	K121N	38, 34	+43, +42
**2**	K121H	<5, <5	+47, +46
**2**	K121C	15, 19	+7, +8
**2**	-	-, -	-, -
**3**	WT	44, 46, 41	-42, -42, -41
**3**	S112Y	<5, <5	+28, +29
**3**	S112L	<5, <5	+37, +36
**3**	S112V	<5, <5	+5, +6
**3**	S112F	<5,<5	-15, -16
**3**	S112M	79, 77	-38, -38
**3**	S112T	<5, <5	-9, -10
**3**	S112Q	-, -	-, -
**3**	S112C	-, -	-, -
**3**	S112R	<5, <5	-60, -61
**3**	S112G	31, 33	-50, -49
**3**	S112H	<5, <5	-50, -51
**3**	S112A	55, 50	-36, -36
**3**	K121R	<5, <5	-9, -10
**3**	K121E	<5, <5	+13, +14
**3**	K121A	5, 7	-13, -13

**3**	K121Y	<5, <5	+23, +22
**3**	K121F	<5, <5	+19, +18
**3**	K121M	5, 7	-15, -15
**3**	K121N	<5, <5	*rac, rac*
**3**	K121H	<5, <5	-9, -10
**3**	K121C	<5, <5	+4, +5
**3**	K121G	<5, <5	-9, -10
**3**	S112D	<5, <5	-45, -46
**3**	S112E	-, -	-, -
**3**	S112K	5, 9	-56, -57

Supporting Table 2. Complete list of control experiments carried out with complex **1** at RT for the synthesis of enantioenriched 2-methoxy-1,1'-binaphthyl **8b**

**Entry**	**Catalyst (Complex 1) 0.10 µmol**	**S112Y-K121E Sav 0.20 µmol**	**Phosphine Ligand without biotin 0.10 µmol**	**[Pd(cinnamyl)(µ-Cl)]$_2$ 0.10 µmol**	**Biotin 0.20 µmol**	**TON**	**ee [%]**
1	yes	-	-	-	-	21, 19	*rac,rac*
2	-	yes	-	-	-	-, -	-, -
3	-	yes	-	yes	-	-, -	-, -
4	yes	yes	-	-	yes	5, 7	-47,- 46
5	-	yes	-	yes	yes	-, -	-, -
6	yes	yes	-	-	-	91, 87	-80, -79
7	-	-	yes	-	-	-, -	-, -
8	-	-	yes	yes	-	-, -	-, -

Supporting Table 3. Complete list of optimization of co-solvent and overall conc. of aryl halide carried out with complex **1** and WT Sav at RT for the synthesis of enantioenriched 2-methoxy-1,1'-binaphthyl **8b**

**Entry**	**Co-solvent**	**ArX (6c) Conc. (mM)**	**TON**	**ee [%]**
1	MeOH	50	70, 74	-52, -52
2	DMSO	50	81, 79	-57, -57
3	THF	50	64, 60	-57, -56
4	Dioxane	50	37, 35	-56, -55
5	DMSO	100	79, 77	-57, -56
5	DMSO	25	52, 48	-55, -55
6	DMSO	12	65, 61	-54, -54
7	DMSO	6	52, 48	-50, -49
8	DMSO	3	46, 44	-49, -50

Supporting Table 4. Complete list of optimization of co-solvent amount, base amount and partial Sav loading carried out with complex **1** and S112Y-K121E Sav at RT for the synthesis of enantioenriched 2-methoxy-1,1'-binaphthyl **8b**

**Entry**	**Pd : Sav tetramer ratio**	**Equivalents base added**	**DMSO in water (%)**	**TON (vs. Pd)**	**ee [%]**
1	1/4	2	10	74, 75	-78, -78
2	2/4	2	10	78, 76	-78, -78
3	3/4	2	10	77, 72	-76, -76
4	4/4	2	10	87, 92	-69, -70

5	2/4	1	10	47, 49	-79, -79
5	2/4	1.5	10	56, 58	-80, -80
6	2/4	3	10	33, 35	-79, -80
7	2/4	5	10	5, 8	-56, -57
8	2/4	2	5	53, 55	-81, -80
9	2/4	2	15	64, 67	-78, -78
10	2/4	2	20	72, 71	-77, -78
11	2/4	2	25	39, 34	-74, -74
12	2/4	2	30	54, 50	-70, -69
13	2/4	2	35	-, -	-, -

Supporting Table 5. Retention times measured on an analytical Chiracel OJ-H column (5μm, 250 x 4.6 mm) at 40 °C with a flow rate: 1.00 ml/min (λ = 254 nm):

Compound	Eluent (hexane/isopropanol)	*(R)* enantiomer	*(S)* enantiomer
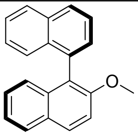 **8b**	95:5	14.28 min	9.21 min
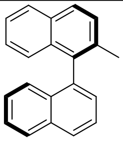 **8a**	95:5	6.58 min	9.49 min
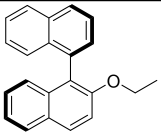 **8c**	95:5	11.12 min	7.66 min
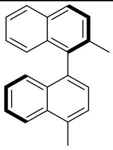 **8d**	90:10	4.75 min	13.27 min

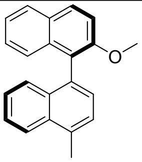 **8e**	90:10	13.68 min	4.79 min
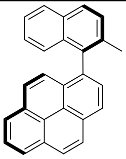 **8f**	90:10	7.62 min	23.38 min
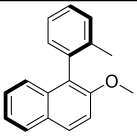 **8g**	95:5	7.48 min	10.46 min
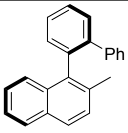 **8h**	95:5	7.60 min	5.02 min

Protein Crystallography

Crystallization. The crystallization screens were carried out by sitting drop vapor diffusion with 1 µl equivolumetric drops of protein and reservoir solution using CrysChem plates that were equilibrated at 20°C. The reservoir solution consisted of 15-25% PEG 1500, 100mM SPG buffer (mixed succinic acid, sodium dihydrogen phosphate and glycine in the ratio 2:7:7; 75% at pH 4 and 25% at pH 10).[6] Good quality crystal could be obtained when drops were streak seeded[7] in low PEG concentration immediately after setting up the drops with crystals growing from high PEG concentration.

The crystals were transferred and soaked for 20 minutes in a cryo-protecting solution containing the biotinylated complex **1**. The cryo-protecting solution consisted of the condition C7 from CryoProtX (Molecular Dimensions)[8] mixed to 20% PEG 1500, 100mM SPG buffer (75% at pH 4 and 25% at pH 10). 0.5uL of ligand **5** at 20mM in 100% DMSO was added to 10µL of the cryo-protecting solution and crystals were flash-cooled in liquid nitrogen.

Data Processing and Refinement. X-ray diffraction data were collected at beam line X06DA at the Swiss Light Source. Data were processed with the XDS software package.[9] The structures

were solved by molecular replacement with the Phaser-MR routine of the Phenix[10] software package, using the 1LUQ structure from the PDB with the waters and ligand removed as a model compound. Structure refinement was performed with the phenix.refine utility.[10] Crystallographic details are given in Table 1.

The Sav S112Y-K121E protein contains two monomers in the asymmetric unit related by non-translational NCS, which form a tetramer by application of the twofold axis. The 12 residues at the N-terminus and the 26 residues at the C-terminus are not resolved. There is strong residual electron density in the 2Fo-Fc difference map in the biotin binding pocket and vestibule. There is a significant peak in the anomalous difference map (8σ) at the interface of the vestibule near Y112, correlating with a strong peak in the density map (12σ); this position was determined to be the Pd atom. Pd atoms display significant anomalous scattering at 1 Å (2e). No other significant peak was found in the anomalous difference map. The density map clearly shows the presence of the phosphine ligand, Pd, and chlorine, but the cinnamyl ligand could not be resolved, likely due to disorder caused by ligand fluxionality. Generation of the initial model and restraints for ligand **1** (without cinnamyl ligand) was done with the eLBOW[11] routine in Phenix. The bond lengths and angles for the phosphine ligand, palladium, and chlorine were referenced from a related structure, [(*t*-Bu_2(4-dimethylanilino)P)PdCl(cinnamyl)],[12] and the biotin and amide linker geometries were optimized with the generalized Amber forcefield in Avogadro v1.1.1[13] (the Pd metal center and ligands were fixed during this step). The amide linker exhibits two H-bonding interactions with the protein backbone: the amide nitrogen exhibits an H-bonding interaction with the sidechain of S88, and the carbonyl oxygen exhibits an H-bonding interaction with the backbone nitrogen of N49.

Wavelength (Å)	1.00
Resolution Range	48.62 - 1.792 (1.856 - 1.792)
Space Group	C 2 2 2_1
Unit Cell	a = 81.368, b = 81.46, c = 90.811<br>$\alpha = \beta = \gamma = 90°$
Total Reflections	329716 (25103)
Unique Reflections	27986 (2541)
Multiplicity	11.8 (9.9)
Completeness (%)	97.60 (89.28)
Mean I/sigma(I)	18.18 (3.20)
Wilson B-factor	18.93
R-merge	0.09055 (0.7346)
R-meas	0.09459
CC1/2	0.999 (0.914)
CC*	1 (0.977)
R-work	0.2231 (0.3084)
R-free	0.2558 (0.3121)
Number of non-hydrogen atoms<br>Macromolecules<br>Ligands<br>Water	1973<br>1863<br>60<br>50
Protein residues	245
RMS(bonds)	0.007
RMS(angles)	1.11
Ramachandran favored	97
Ramachandran allowed	0
Ramachandran outliers	0
Clashscore	2.73
Average B-factor<br>Macromolecules<br>Ligands<br>Solvent	24.20<br>23.80<br>36.10<br>25.00

Statistics for the highest-resolution shell are shown in parenthesis.

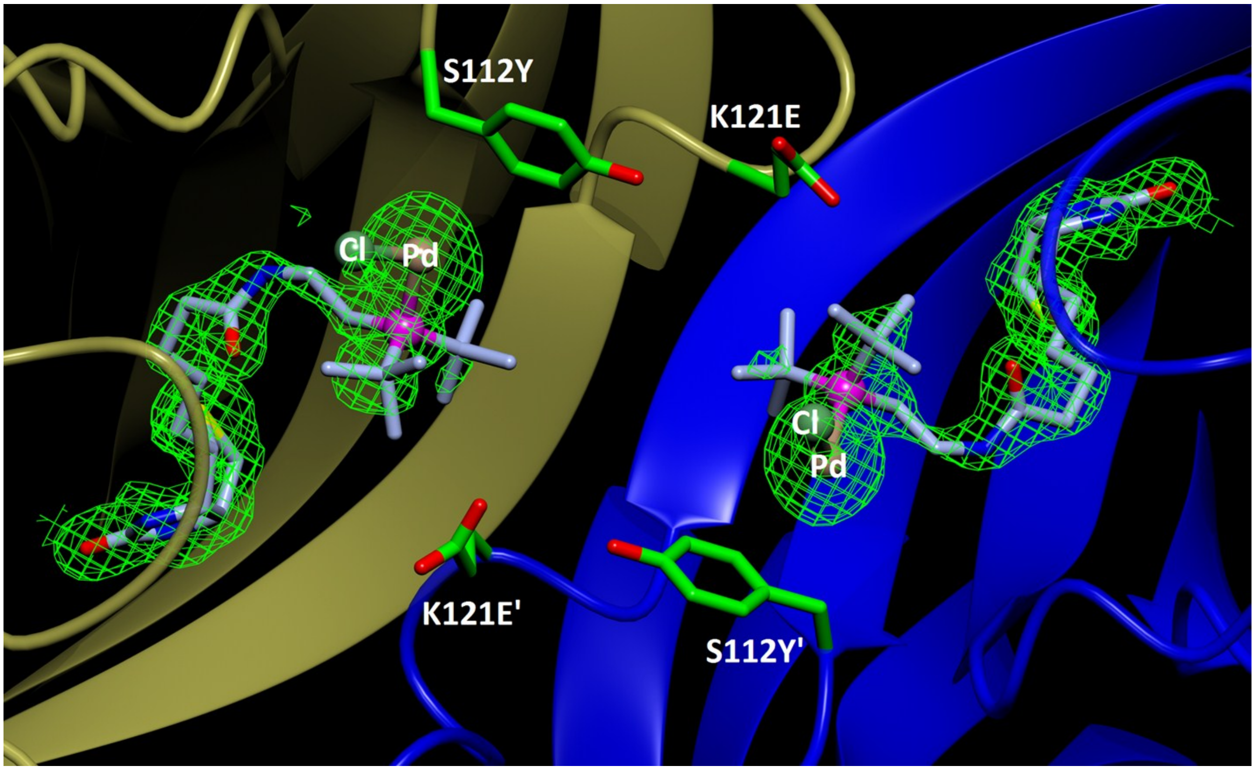

Supporting Figure 5. Close-up view of the X-ray structure of 1 · S112Y-K121E Sav. The two monomeric chains (blue and gold) and biotinylated complexes are related by a twofold symmetry axis. The electron density around **1** (green) was generated from a 2Fo-Fc map (0.5 electrons/Å^3).

Spectra for complex 1:

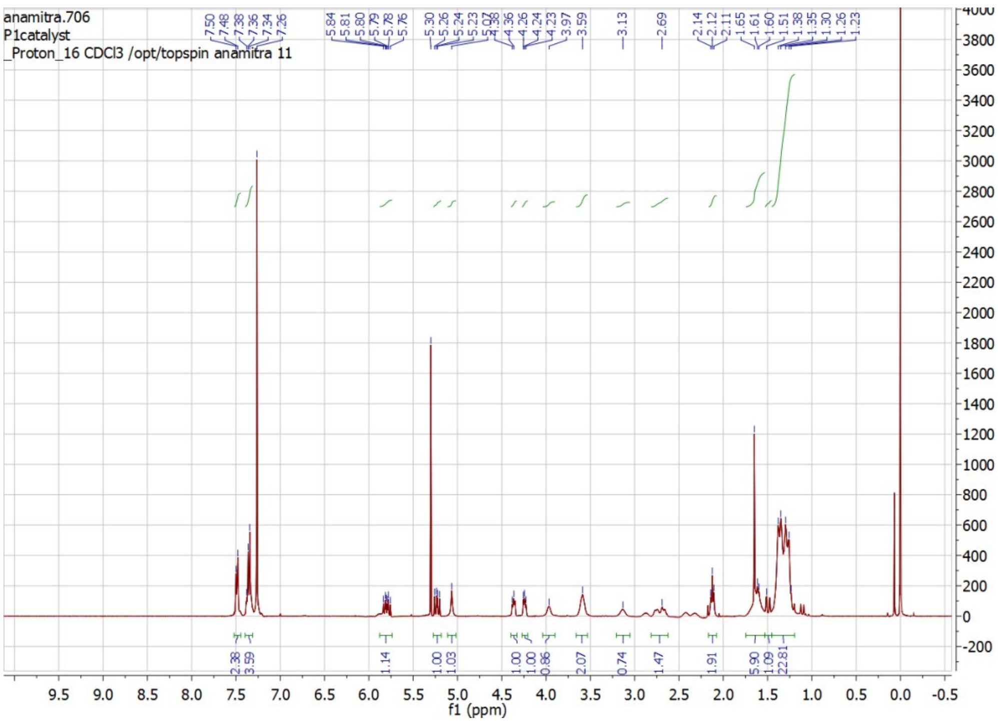

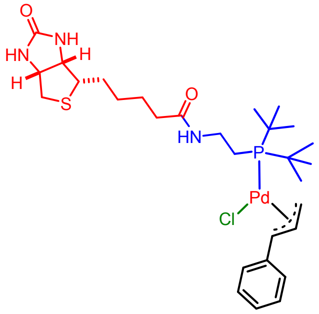

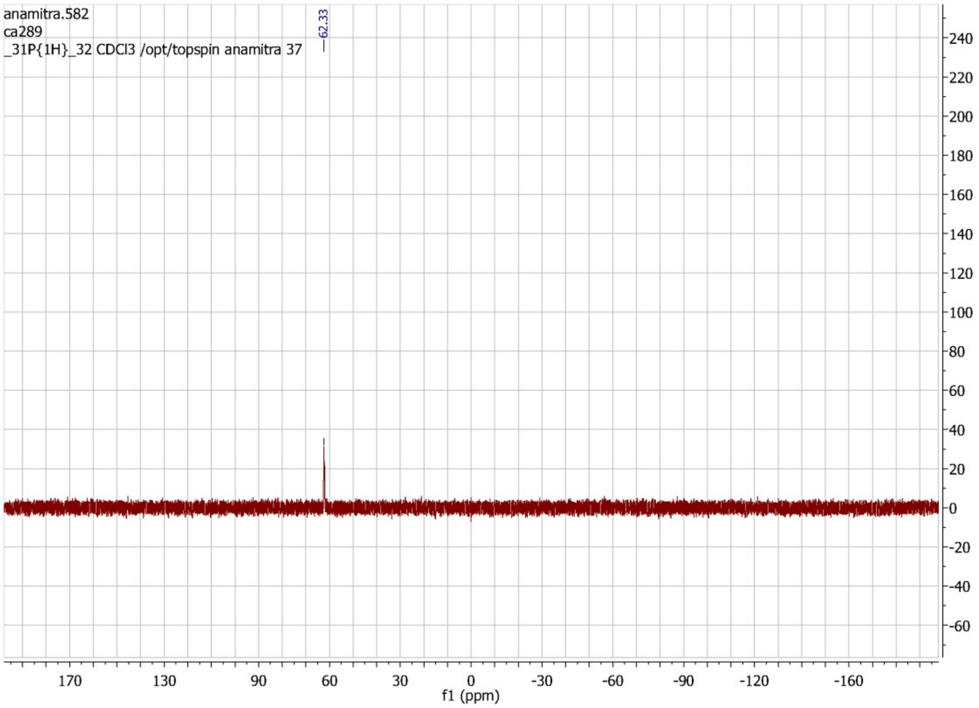

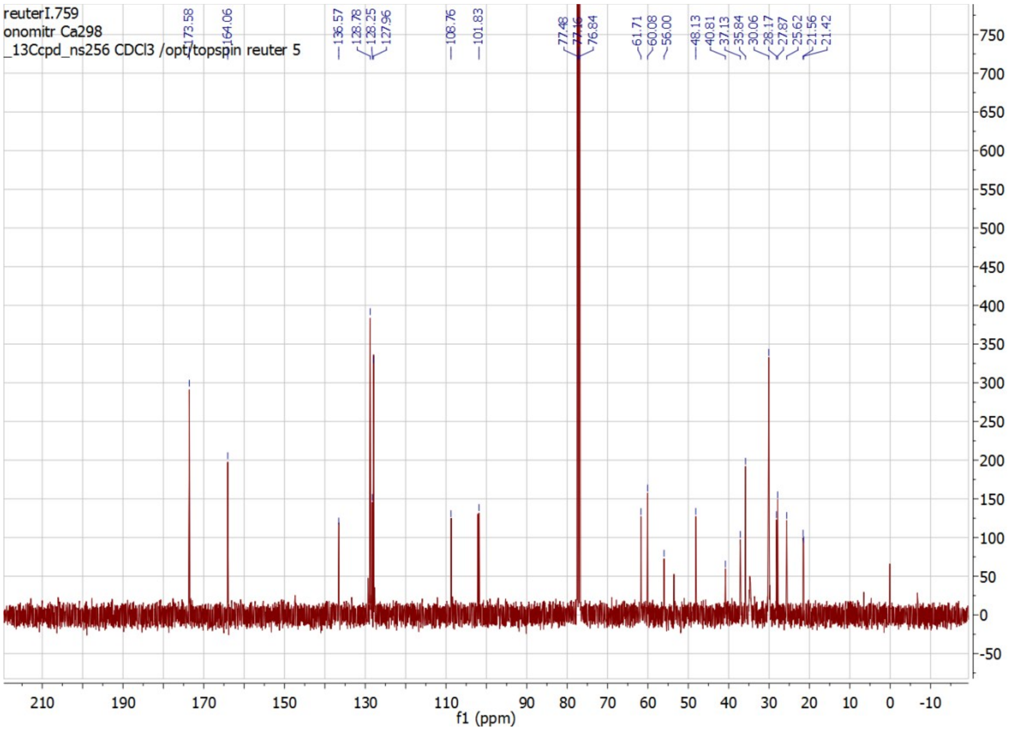
reuterI.759
onomitr Ca298
_13Ccpd_ns256 CDCl3 /opt/topspin reuter 5
173.58
164.06
136.57
128.78
128.25
127.96
108.76
101.83
77.48
77.16
76.84
61.71
60.08
56.00
48.13
40.81
37.13
35.84
30.06
28.17
27.87
25.62
21.56
21.42
f1 (ppm)

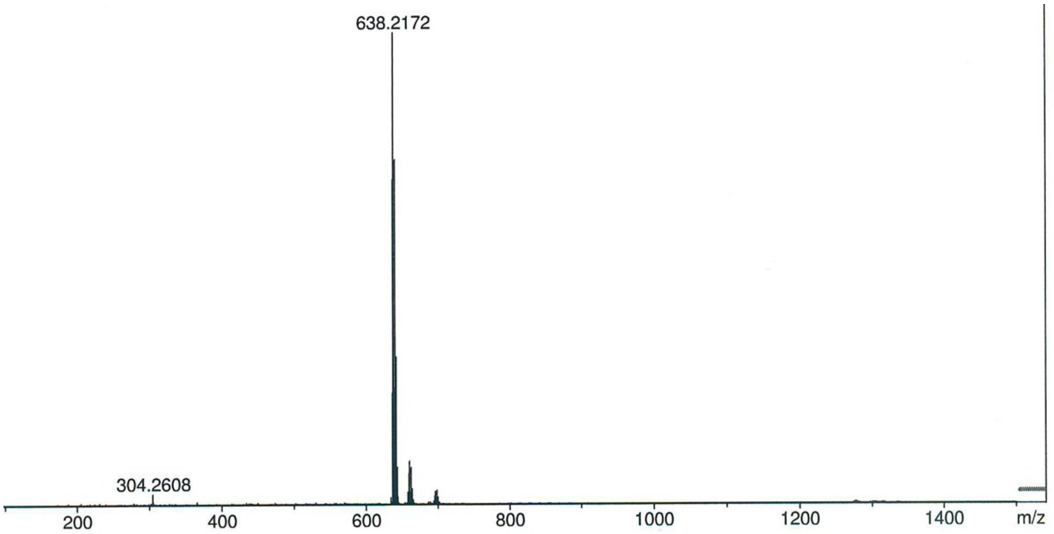
638.2172
304.2608
m/z

Spectra for complex 2:

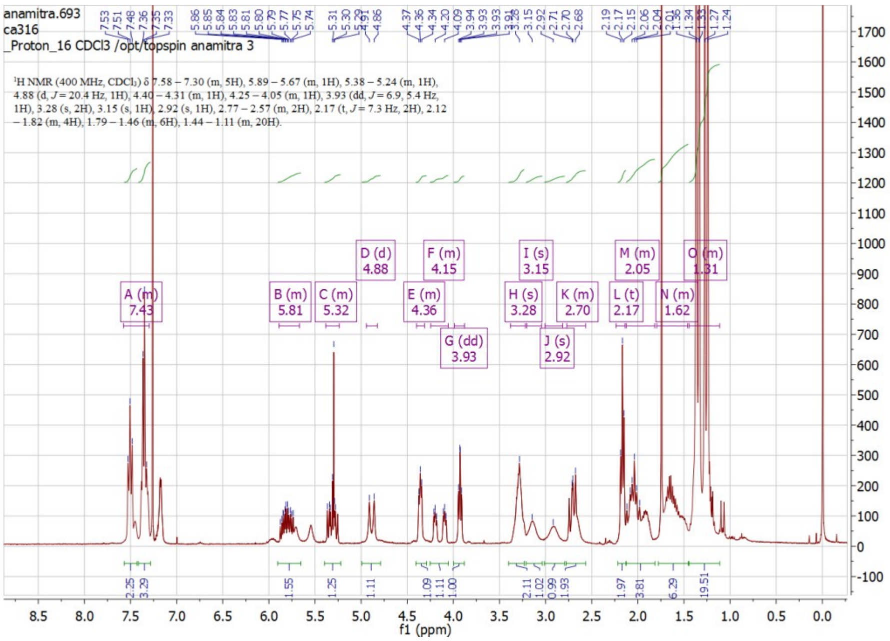

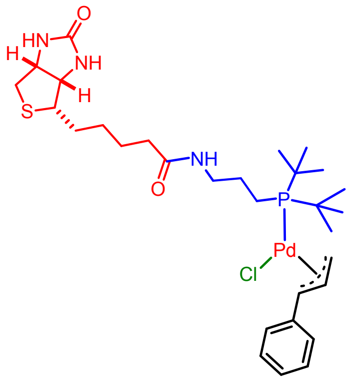

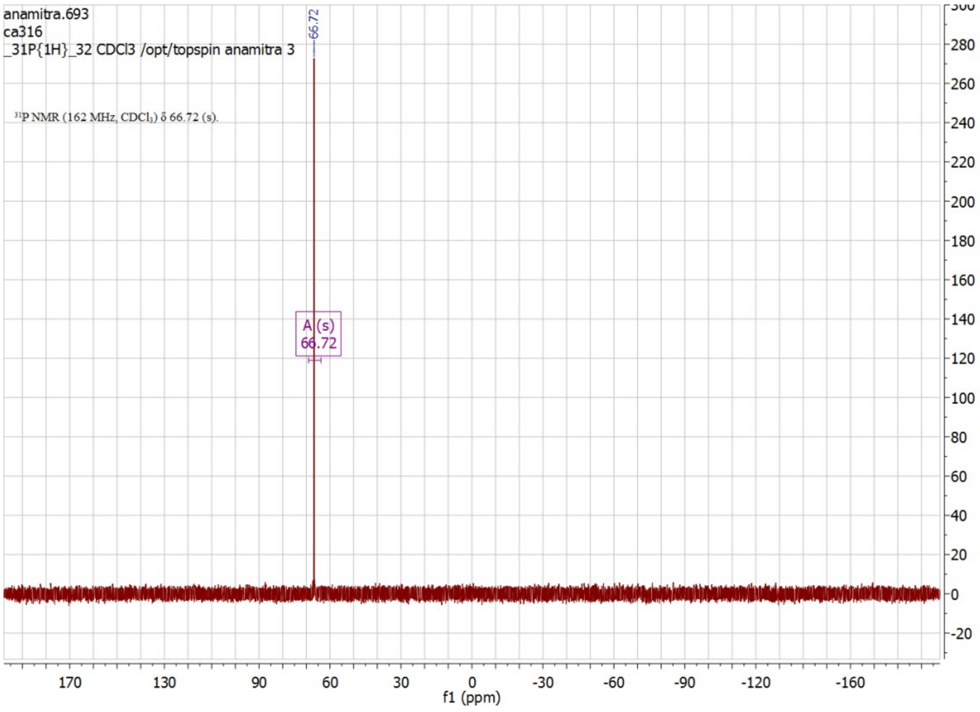

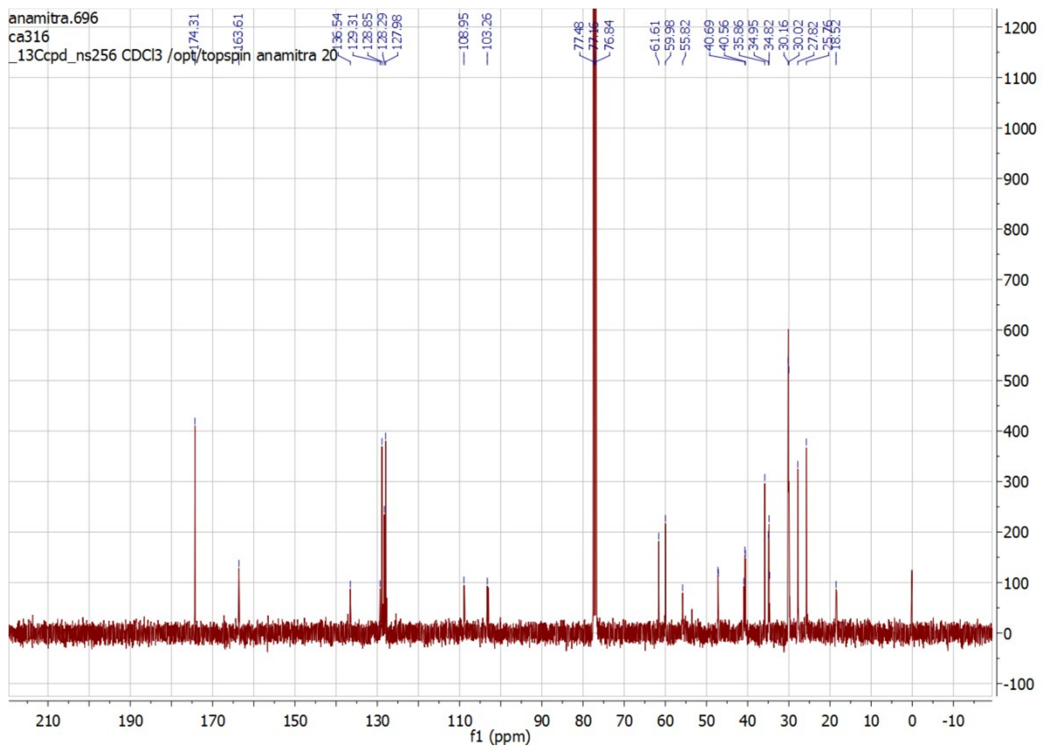
anamitra.696
ca316
_13Ccpd_ns256 CDCl3 /opt/topspin anamitra 20
174.31
163.61
136.54
129.31
128.85
128.29
127.98
108.95
103.26
77.48
76.84
61.61
59.98
55.82
40.69
40.56
35.86
34.95
34.82
30.16
30.02
27.82
18.52
f1 (ppm)

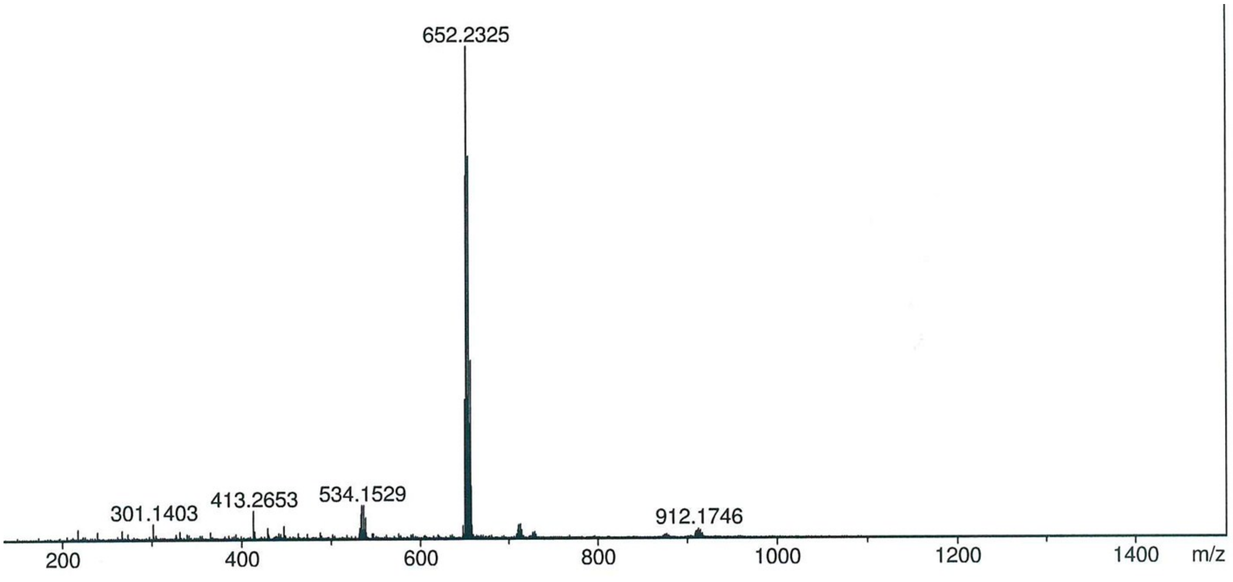
652.2325
301.1403
413.2653
534.1529
912.1746
m/z

Spectra for complex 3:

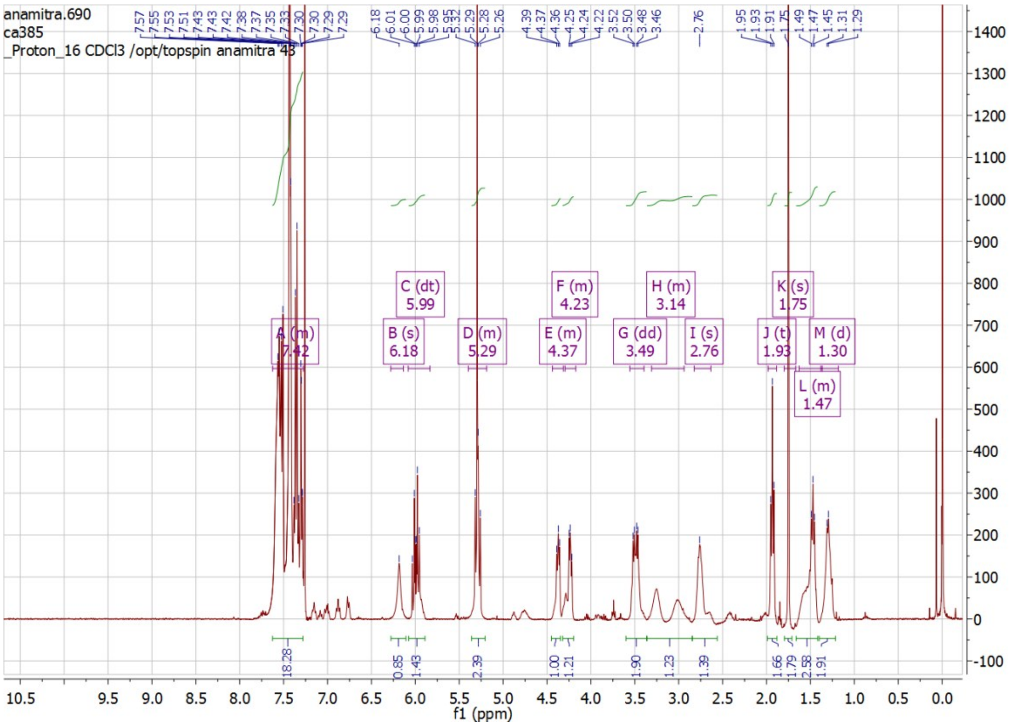

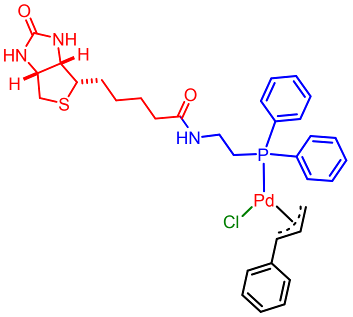

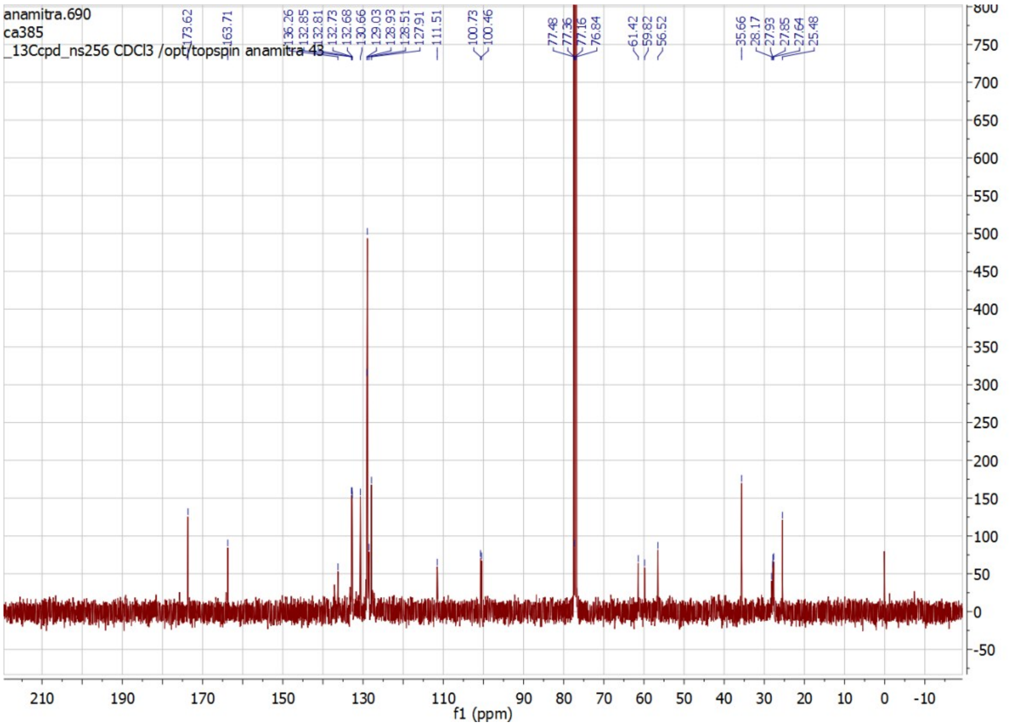

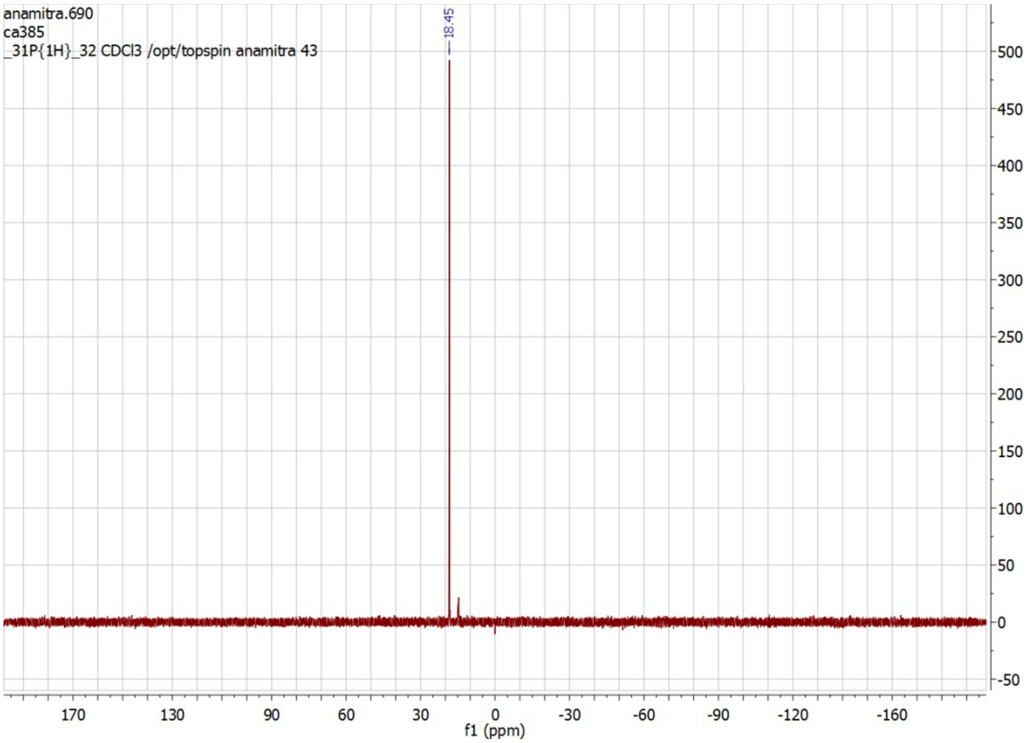
anamitra.690
ca385
_31P{1H}_32 CDCl3 /opt/topspin anamitra 43
18.45
500
450
400
350
300
250
200
150
100
50
0
-50
170
130
90
60
30
0
-30
-60
-90
-120
-160
f1 (ppm)

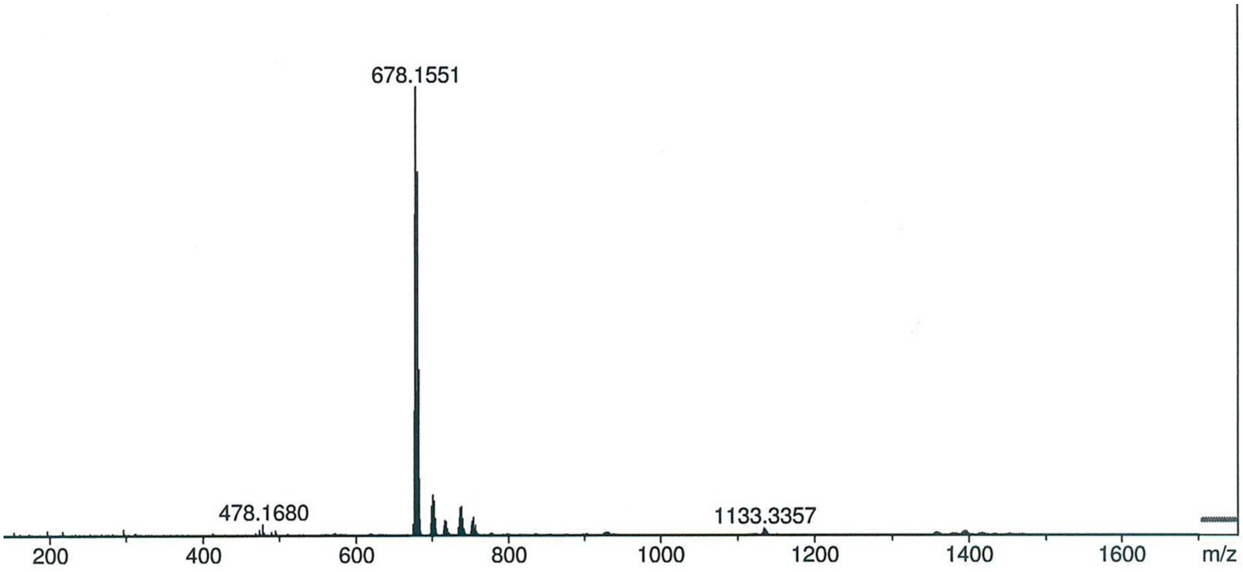
678.1551
478.1680
1133.3357
200
400
600
800
1000
1200
1400
1600
m/z

Spectra for complex 4:

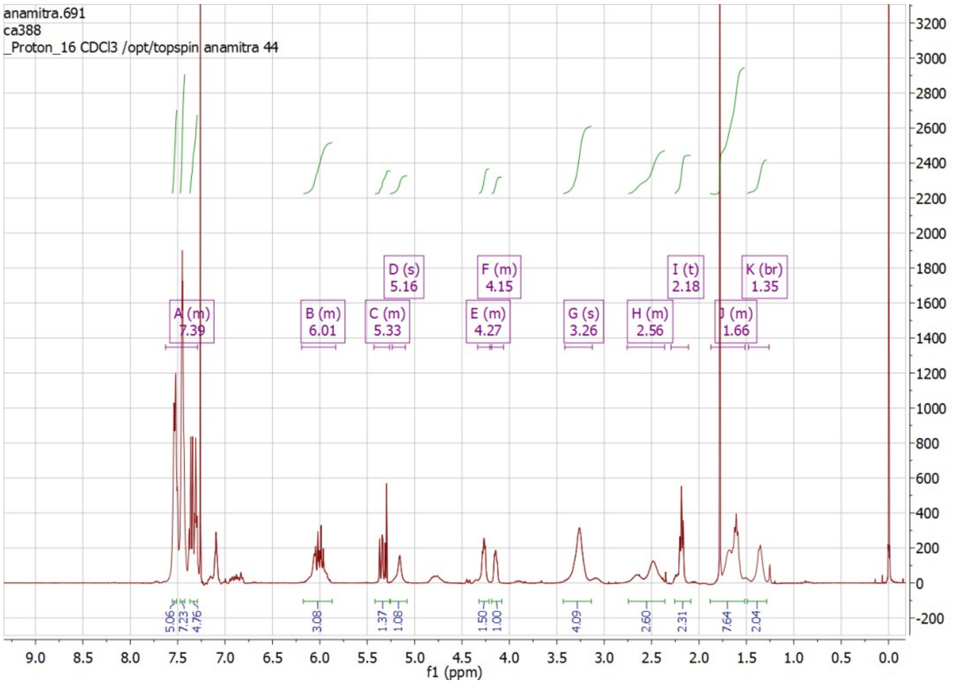

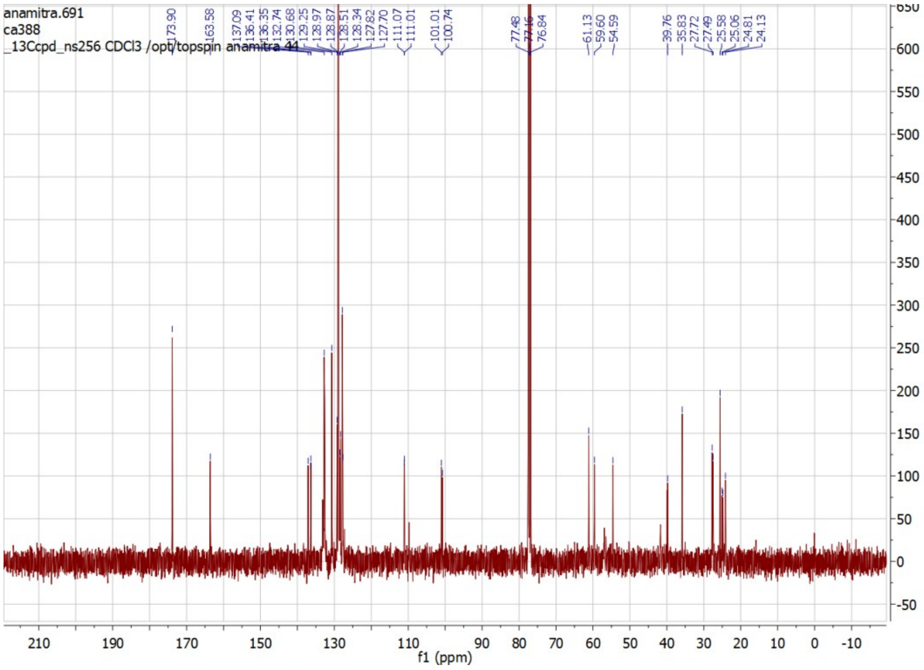

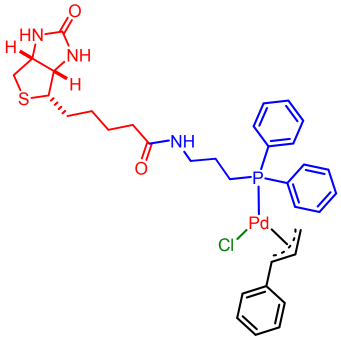

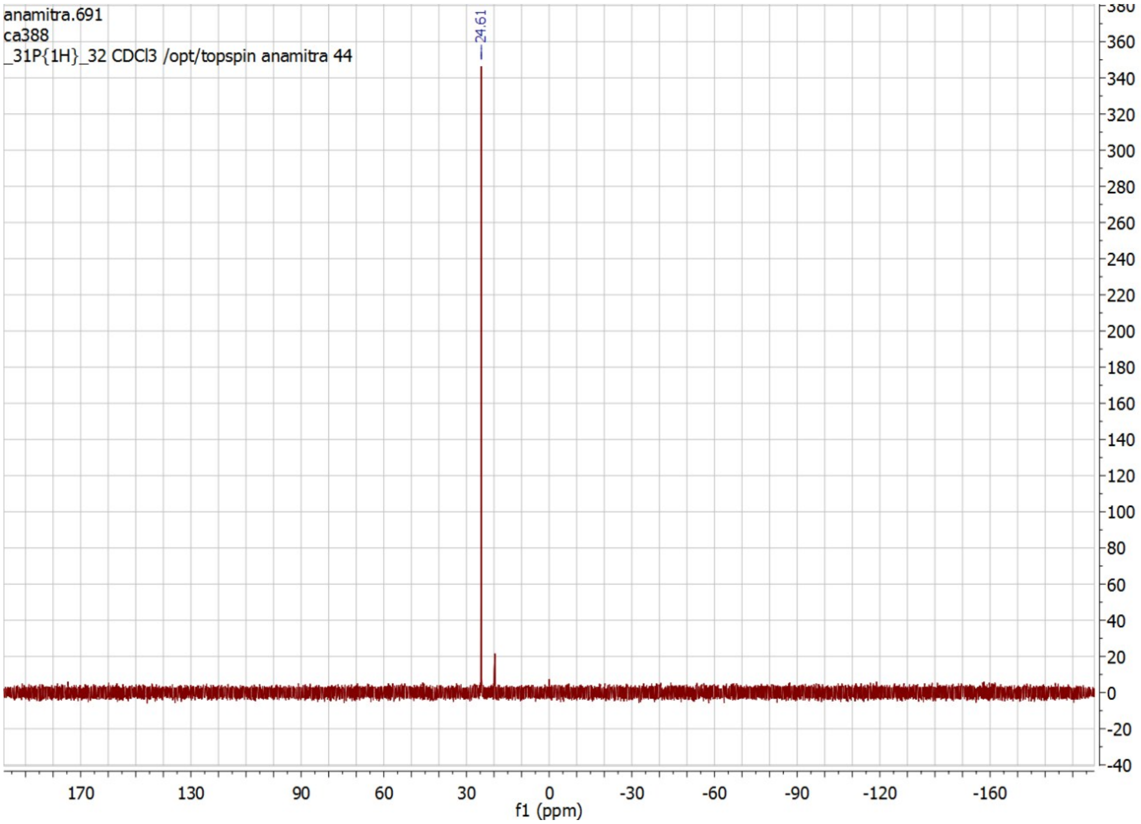
anamitra.691
ca388
_31P{1H}_32 CDCl3 /opt/topspin anamitra 44
24.61
f1 (ppm)

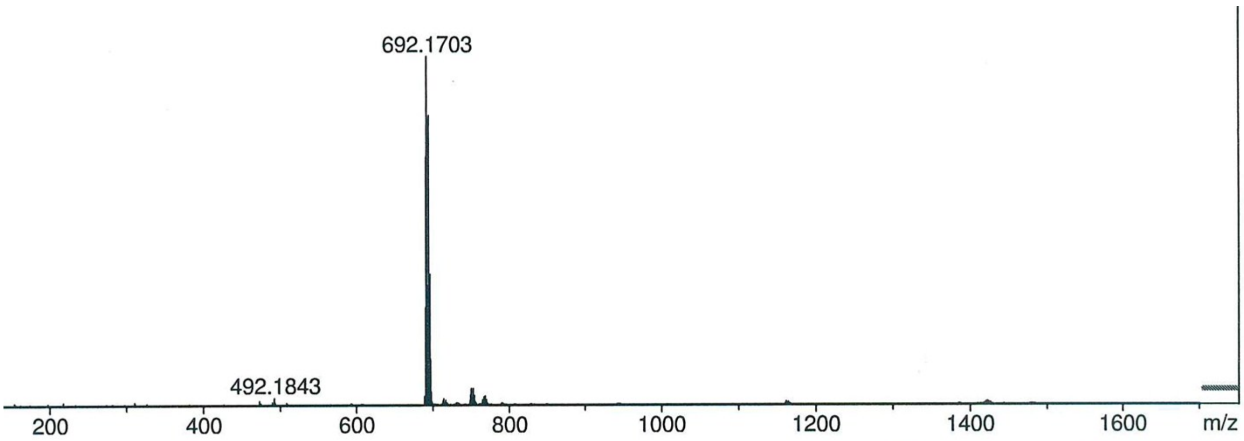
692.1703
492.1843
m/z

Spectra for complex 5:

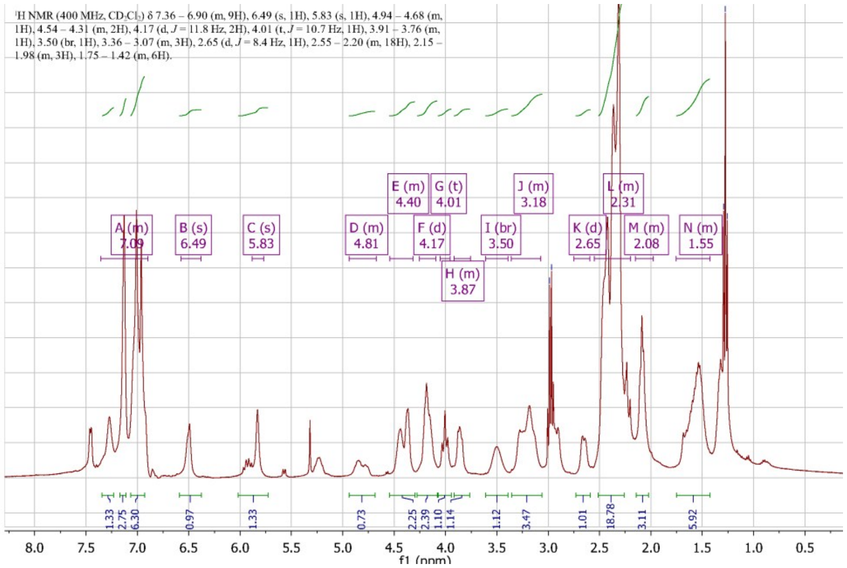

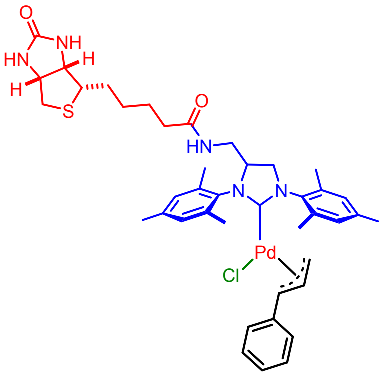

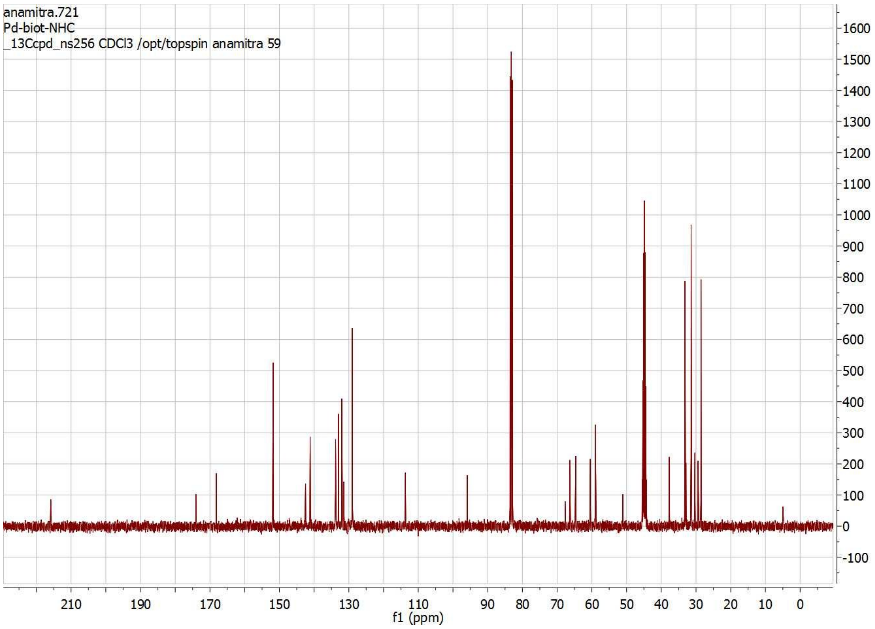

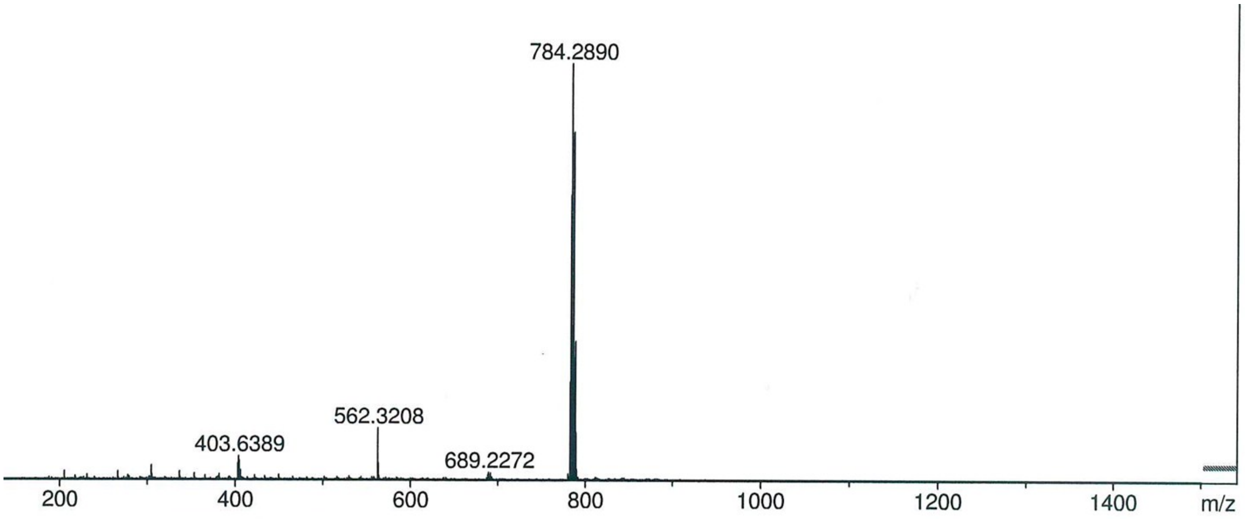

Literature Cited:

[1] G. Klein, N. Humbert, J. Gradinaru, A. Ivanova, F. Gilardoni, U. E. Rusbandi and T. R. Ward, *Angew. Chem. Int. Ed.* 2005, **44**, 7764.

[2] A. Reizelman, S. C. M. Wigchert, C. Bianco and B. Zwanenburg, *Org. Biomol. Chem.* 2003, **1**, 950.

[3] J. P. Jordan and R. H. Grubbs, *Angew. Chem. Int. Ed.* 2007, **46**, 5152.

[4] E. A. Bayer, S. Ehrligh-Rogozinski and M. Wilchek, *Electrophoresis* 1996, **17**, 1319.

[5] N. Humbert, A. Zocchi and T. R. Ward, *Electrophoresis* 2005, **26**, 47.

[6] J. Newman, *Acta Cryst.* 2004, **D60**, 610.

[7] E. A. Stura, and I. A. Wilson, *J. Cryst. Growth.* 1991, **110**, 270.

[8] L. Vera and E. A. Stura, *Cryst. Growth Des.* 2013, **13**, 1878.

[9] W. Kabsch, *Acta Cryst.* 2010, **D66**, 125.

[10] P. D. Adams, P. V. Afonine, G. Bunkoczi, V. B. Chen, I. W. Davis, N. Echols, J. J. Headd, L. W. Hung, G. J. Kapral, R. W. Grosse-Kunstleve, A. J. McCoy, N. W. Moriarty, R. Oeffner, R. J. Read, D. C. Richardson, J. S. Richardson, T. C. Terwilliger and P. C. Zwart, *Acta Cryst.* 2010, **D66**, 213.

[11] N. W. Moriarty, R. W. Grosse-Kunstleve and P. D. Adams, *Acta Cryst.* 2009, **D65**, 1074.

[12] A. Chartoire, M. Lesieur, A. M. Z. Slawin, S. P. Nolan and C. S. J. Cazin, *Organometallics* 2005, **30**, 4432.

[13] M. D. Hanwell, D. C. Curtis, D. C. Lonie, T. Vandermeersch, E. Zurek and G. R. Hutchison, *J. Cheminformatics* 2012, **4**, 17.
